# Identification of a New Cholesterol‐Binding Site within the IFN‐*γ* Receptor that is Required for Signal Transduction

**DOI:** 10.1002/advs.202105170

**Published:** 2022-02-15

**Authors:** Ornella Morana, Jon Ander Nieto‐Garai, Patrik Björkholm, Jorge Bernardino de la Serna, Oihana Terrones, Aroa Arboleya, Dalila Ciceri, Iratxe Rojo‐Bartolomé, Cédric M. Blouin, Christophe Lamaze, Maier Lorizate, Francesc‐Xabier Contreras

**Affiliations:** ^1^ Instituto Biofisika (UPV/EHU, CSIC) University of the Basque Country (UPV/EHU) Barrio Sarriena s/n Leioa E‐48940 Spain; ^2^ Fundación Biofísica Bizkaia/Biofisika Bizkaia Fundazioa (FBB) University of the Basque Country (UPV/EHU) Barrio Sarriena s/n Leioa E‐48940 Spain; ^3^ Department of Biochemistry and Molecular Biology Faculty of Science and Technology University of the Basque Country (UPV/EHU) Barrio Sarriena s/n Leioa E‐48940 Spain; ^4^ Center for Biomembrane Research Department of Biochemistry and Biophysics Stockholm University Stockholm SE‐106 91 Sweden; ^5^ Science for Life Laboratory Stockholm University Solna SE‐171 21 Sweden; ^6^ National Heart and Lung Institute Faculty of Medicine Imperial College London South Kensington Sir Alexander Fleming Building London SW7 2AZ UK; ^7^ Central Laser Facility Rutherford Appleton Laboratory MRC‐Research Complex at Harwell Science and Technology Facilities Council Harwell OX11 0QX UK; ^8^ NIHR Imperial Biomedical Research Centre London SW7 2AZ UK; ^9^ Institut Curie ‐ Centre de Recherche PSL Research University Membrane Mechanics and Dynamics of Intracellular Signaling Laboratory Paris 75248 France; ^10^ Institut National de la Santé et de la Recherche Médicale (INSERM) Paris U1143 France; ^11^ Centre National de la Recherche Scientifique (CNRS) UMR 3666 Paris 75248 France; ^12^ IKERBASQUE Basque Foundation for Science Bilbao 48011 Spain

**Keywords:** cholesterol, interferon gamma receptors, lipid nanodomains, protein–lipid interactions, signal transduction

## Abstract

The cytokine interferon‐gamma (IFN‐*γ*) is a master regulator of innate and adaptive immunity involved in a broad array of human diseases that range from atherosclerosis to cancer. IFN‐*γ* exerts it signaling action by binding to a specific cell surface receptor, the IFN‐*γ* receptor (IFN‐*γ*R), whose activation critically depends on its partition into lipid nanodomains. However, little is known about the impact of specific lipids on IFN‐*γ*R signal transduction activity. Here, a new conserved cholesterol (chol) binding motif localized within its single transmembrane domain is identified. Through direct binding, chol drives the partition of IFN‐*γ*R2 chains into plasma membrane lipid nanodomains, orchestrating IFN‐*γ*R oligomerization and transmembrane signaling. Bioinformatics studies show that the signature sequence stands for a conserved chol‐binding motif presented in many mammalian membrane proteins. The discovery of chol as the molecular switch governing IFN‐*γ*R transmembrane signaling represents a significant advance for understanding the mechanism of lipid selectivity by membrane proteins, but also for figuring out the role of lipids in modulating cell surface receptor function. Finally, this study suggests that inhibition of the chol‐IFN*γ*R2 interaction may represent a potential therapeutic strategy for various IFN‐*γ*‐dependent diseases.

## Graphical abstract

This work uncovers the role of cholesterol as the molecular determinant controlling interferon‐gamma receptor (IFN‐γR) activity. Through a multidisciplinary approach the structural signature within the IFN‐γR required for cholesterol binding is identified. Binding of cholesterol is critical for receptor plasma membrane compartmentalization, heterodimerization, and signal transduction. This work identifies a therapeutic target for prevention and treatment of IFN‐γ dependent diseases.

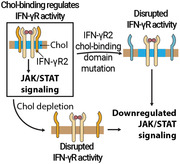

## 1 Introduction

The proper functioning of cell surface receptors critically depends on how their transmembrane domains (TMDs) are embedded and interact with their surrounding lipids along the plasma membrane (PM). This nanoscale PM organization plays a crucial role in controlling spatio‐temporal protein lateral compartmentalization and providing a perfect environment for protein functioning by trapping membrane receptors in defined lipid nanodomains.^[^ ^1^, ^2^ ^]^ A specific subset of these lipid nanodomains are chol‐ and sphingolipid‐ (SP) enriched nanostructures (often referred to as lipid rafts), that exhibit an intrinsic ability to assemble/disassemble rapidly and dynamically in a process tightly related to the activation of membrane receptors.^[^ ^2^, ^3^, ^4^, ^5^, ^6^, ^7^ ^]^ Over the last decades, a sizeable research effort has been conducted to identify different cellular roles for lipid nanodomains and understand how lipid nanoscale organization influences transmembrane protein functions at the PM.^[^ ^1^, ^5^, ^6^, ^8^ ^]^ Transmembrane protein features such as palmitoylation, TMD length, and accessible surface area (ASA) have been postulated to play an essential role in protein nanodomain recruitment.^[^ ^9^ ^]^ However, results obtained with different methods lead to controversial results,^[^ ^10^ ^]^ and the precise molecular mechanisms driving protein partition/exclusion into lipid nanodomains in living cells are still elusive. An alternative mechanism to maintain or exclude membrane proteins from lipid nanodomains is by intramembrane protein–lipid interactions between specific lipids enriched in these nanostructures and the TMD of the membrane protein. While an extended body of research has been conducted to decipher the role of defined lipids as cofactors regulating and stabilizing single‐ and multi‐span transmembrane proteins,^[^ ^11^, ^12^ ^]^ to date, a direct role of defined lipids in transmembrane receptors targeting and retention into membrane nanodomains has not been decrypted. This pending question is particularly intriguing for transmembrane signaling receptors that could be potential targets for drug developers to home in since they play a key role in the regulation of a wide range of signaling processes associated with various common human diseases.^[^ ^13^ ^]^ To understand the molecular mechanism underpinning signaling, transmembrane receptor lateral segregation, and partition into lipid nanodomains, we studied the interferon‐gamma receptor (IFN‐*γ*R). This receptor is a heterotetramer composed of two IFN‐*γ*R1 and two IFN‐*γ*R2 chains, whose lateral segregation and partition into lipid nanodomains are essential prerequisites for receptor conformational change and transmembrane signal transduction.^[^ ^14^ ^]^ IFN‐*γ*R activates the Janus‐activated tyrosine kinase (JAK)/signal transducer and activator of transcription (STAT) signaling pathway.^[^ ^15^, ^16^ ^]^ In the last years, there has been a renewed interest in interferon‐*γ* (IFN‐*γ*) biology due to its regulatory effects on immune evasion by controlling immune checkpoint genes that drive tumorigenesis^[^ ^17^, ^18^, ^19^ ^]^ as well as regulating atherosclerosis^[^ ^20^ ^]^ and autoimmunity.^[^ ^21^ ^]^ Thus, it is critical to understand the precise molecular mechanisms controlling the IFN‐*γ* signaling axis to develop novel strategies to fight diseases that are under the control of this signaling pathway.

In this study, using a multidisciplinary approach, we have identified a new chol‐binding domain in the TMD of the IFN‐*γ*R2 chain. We further show that binding of chol to the receptor governs receptor spatiotemporal lateral segregation into lipid nanodomains, controls receptor oligomerization, and transmembrane signal transduction. By blocking chol biosynthesis, we provide evidence of the effect of downregulating the IFN‐*γ* signaling axis in the cell surface expression of the immune checkpoint PD‐L1 in various types of cancer cells. Finally, bioinformatic analyses predict that the lineal signature sequence represents a conserved chol‐binding crevice in a variety of mammalian single‐ and multi‐span membrane proteins mainly localized in the PM. Notably, a high number of new candidates correspond to ion channels and G‐protein coupled receptors (GPCRs). Taken together, our results prove that chol has a previously unrecognized role as a molecular switch regulating the signal transduction activity of the IFN‐*γ*R and highlights the IFN‐*γ*R/chol tandem as a promising therapeutic target for the treatment and prevention of various IFN‐*γ* signaling dependent diseases.

## 2 Results

### 2.1 IFN‐*γ*R Interacts with Chol and Sphingolipids in Living Cells

**Figure 1:**
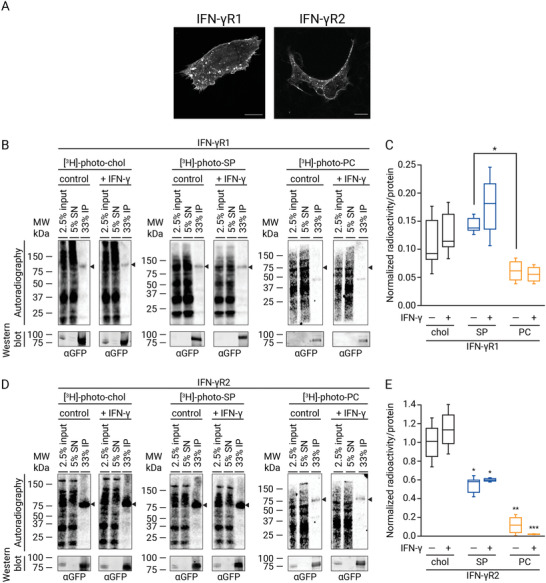
IFN‐*γ*R interacts with chol and SP but not with PC in vivo prior to and following IFN‐*γ* stimulation. A) Fluorescence microscopy images of transiently expressed full‐length IFN‐*γ*R1‐ and IFN‐*γ*R2‐GFP tagged proteins localization in CHO cells. Scale bar = 20 µm. B,C) In vivo photoaffinity labeling of IFN‐*γ*R1 using tritiated and photolabile chol, SP, and PC. In (B) CHO cells transiently expressing IFN‐*γ*R1‐GFP and IFN‐*γ*R2‐Luc constructs were treated with 100 µCi (3 µm), 60 *μ*Ci (2 µm) of the bifunctional chol and SP analogues for 6 h, respectively. For PC labeling, cells were treated with 50 *μ*Ci (2 µm) [^3^H]‐choline combined with 100 µm 10‐ASA for 6 h. Before ultraviolet irradiation, cells were treated for 5 min with IFN‐*γ* (1000 U mL^−1^) or vehicle. Finally, cells were lysed, subjected to immunoprecipitation against the GFP epitope and input, supernatant (SN), and immunoprecipitation (IP) were analyzed by western blot and digital autoradiography (*n* = 3 independents experiments). C) Quantification of immunoprecipitated radioactivity/IFN‐*γ*R1 protein for chol, SP, and PC binding (data are the mean ± SD; *n* = 3 independent experiments). Data were normalized to IFN‐*γ*R2WT‐chol interactions. D,E) In vivo photoaffinity binding IFN‐*γ*R2WT with bifunctional chol, SP, and PC lipids in living cells. D) CHO cells transiently expressing the IFN‐*γ*R2‐GFP/IFN‐*γ*R1‐Luc constructs were treated and handled as described in (B) (*n* = 3 independents experiments). E) Quantification of immunoprecipitated radioactivity/IFN‐*γ*R2 protein for chol, SP, and PC binding (data are the mean ± SD; *n* = 3 independent experiments. The line on each of the boxes represents the median for that particular data set). Arrow, expected protein sizes. Statistical significances were determined with one‐way ANOVA Bonferroni‘s multiple comparison test, and is represented compared to IFN‐*γ*R2WT‐chol interactions (***p < 0.001; ns: not significant).

We first carried out in vivo photoaffinity experiments using radioactive and photoactivatable lipids^[^ ^22^, ^23^ ^]^ to characterize the binding of lipids to IFN‐*γ*R in their native environment. We used bifunctional sphingosine (Sph), a lipid precursor of sphingolipids (SP), and bifunctional chol as lipid nanodomains markers (Figure S1A,B, Supporting Information). As a non‐nanodomain lipid marker, we combined [^3^H]‐choline with a diazirine‐containing fatty acid (10‐ASA). These lipid precursors enter into the phosphatidylcholine synthesis pathway leading to the successful biosynthesis of bifunctional PC species in cells as previously reported (Figure S1C, Supporting Information).^[^ ^23^ ^]^ After lipid photolabile addition and incubations, cells were UV‐irradiated, thereby creating a covalent cross‐linking between the lipid analogue and the proteins at a distance below 3 Å (Figure S1D, Supporting Information).^[^ ^12^, ^24^ ^]^ We initially validated the technique using two well‐characterized membrane proteins known to be inserted or excluded from lipid nanodomains. As marker and non‐marker of lipid nanodomains, Caveolin‐1 (Cav‐1)^[^ ^23^, ^25^ ^]^ and Tranferrin receptor (TfR)^[^ ^26^ ^]^ were selected, respectively. As observed in Figure S2A, Supporting Information, TfR strongly interacts with PC while no interaction with chol was observed. In the case of Cav‐1, no direct interaction of Cav‐1 with PC was observed (Figure S2B, Supporting Information) as previously reported.^[^ ^23^ ^]^ On the contrary, a tight interaction with the two lipid nanodomains markers (chol and SP) in vivo was observed with Cav‐1 (Figure S2B, Supporting Information). Once the methodology was validated, we started to study the in vivo lipid environment of the two IFN‐*γ*R chains. IFN‐*γ*R1 or IFN‐*γ*R2 chains were fused to green fluorescent protein (GFP). They showed a proper cellular distribution (**Figure** **1**A) and were used to investigate the in vivo binding of lipids to the receptor in unstimulated versus stimulated cells.^[^ ^14^ ^]^ To this end, cells expressing the tandem IFN‐*γ*R1‐GFP/IFN*γ*‐R2‐RLuc or IFN*γ*‐R2‐GFP/IFN*γ*‐R1‐RLuc for fully functionalized human receptor in CHO cells were fed with the different bifunctional lipids. Then, cells were UV‐light irradiated to covalently link photoactivatable lipids to proteins nearby, lysed, and subjected to immunoprecipitation using an anti‐GFP antibody. Lipid cross‐linking revealed a noticeable interaction of the IFN‐*γ*R1 chain with both chol and SP, with a two‐ and three‐fold enrichment in radioactivity recovery per mol of protein for chol and SP, respectively, compared to PC in resting and IFN‐*γ* stimulated cells (Figure 1B,C). Strikingly, when the IFN‐*γ*R2 chain was investigated, an even higher interaction with chol and SP with marginal PC labeling was observed, with a ten‐ and five‐fold enrichment factor for chol and SP compared to PC in both unstimulated and stimulated cells, respectively (Figure 1D,E). Thus, in living cells, the IFN‐*γ*R is confined into chol‐ and SP‐enriched nanodomains independently of activation by IFN‐*γ*. Since the IFN‐*γ*R2 chain showed a high propensity to interact with chol and SP in vivo (Figure 1D,E), next, we focused on investigating the specificity of these interactions in living cells. To this end, we performed competition experiments by administering separately to cells the bifunctional analogs and increasing amounts of the corresponding competing native lipids. As observed in Figure S2C,D, Supporting Information, the cross‐linking between [^3^H]‐photo‐chol and IFN‐*γ*R2 was competed in a dose‐dependent manner when unmodified native chol was included in the incubation step. This competition effect was not observed when the same experiment was performed with [^3^H]‐photo‐SP and increasing amounts of native SP (Figure S2E,F, Supporting Information). These results strongly suggest that the [^3^H]‐photo‐chol probe specifically reports the direct interaction of chol with the IFN‐*γ*R2 chain.

### 2.2 No Role for CRAC Motif in Chol Binding to IFN‐*γ*R2

Guided by the in vivo photoaffinity results, we hypothesized that direct binding of specific lipids to the IFN‐*γ*R transmembrane domain (IFN‐*γ*RTMD) could control receptor partition/exclusion into lipid nanodomains and thus receptor function. To test this hypothesis, we initially focused our efforts on screening for previously described lipid‐binding domains within the IFN‐*γ*R2. Several chol‐binding domains have been described in the past that share a common pattern of positive, aromatic, and hydrophobic amino acid residues.^[^ ^27^ ^]^ Among them, the cholesterol recognition amino acid consensus (CRAC) motif with a linear signature [(L/V)‐X_1–5_‐Y‐X_1–5_‐(R/K)] found in a large number of integral membrane proteins is the best described.^[^ ^27^, ^28^ ^]^ Bioinformatic studies identified a CRAC domain localized proximal to the C‐terminal moiety of the IFN‐*γ*R2TMD (Figure S3A, Supporting Information). To investigate the putative role of the CRAC domain in chol binding, we performed site‐directed mutagenesis of the key amino acids of the CRAC motif and tested the mutant by in vivo photoaffinity experiments (Figure S3B, Supporting Information). Cells expressing IFN‐*γ*R2‐CRAC‐GFP mutated protein (Figure S3C, Supporting Information) were fed in vivo with [^3^H]‐photo‐chol, UV‐irradiated, and protein immunoprecipitated as before. As observed in Figure S3D, Supporting Information, the IFN‐*γ*R2‐CRAC mutant did not lose its ability to bind chol in vivo. The photoaffinity results obtained ruled out any implication of the predicted CRAC domain in the direct in vivo binding of chol to IFN‐*γ*R2.

### 2.3 Identifying a New Chol‐Binding Motif in IFN‐*γ*R2TMD

**Figure 2:**
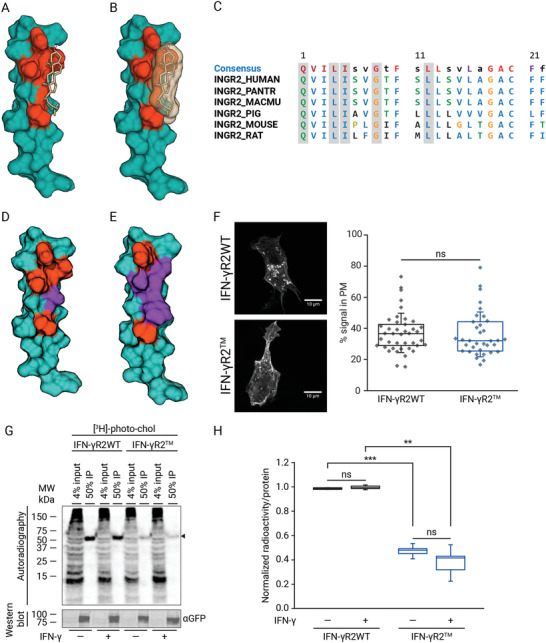
A new conserved chol‐binding domain within the IFN‐*γ*R2‐TMD. A,B) Molecular docking prediction of the chol‐IFN‐*γ*R2TMD interaction. In (A) amino acids forming the binding groove are highlighted in the 3D minimal energy structure of the IFN‐*γ*RTMD. Dark Green, IFN‐*γ*R2TMD + 3 N‐terminal juxtamembrane residues; orange, chol‐binding pocket; light‐brown, chol. B) Chol space‐filling occupancy within the binding domain highlighted in orange as described in (A) in a 3D minimal energy structure of the IFN‐*γ*R2TMD. C) Conservation analysis of the chol‐binding domain across IFN‐*γ*R2TMD in different species. The conserved residues are shaded in red and amino acids forming the chol‐binding pocket are boxed in blue. D,E) Energy‐minimized structure of IFN‐*γ*R2TMD mutants. D) IFN‐*γ*R2^G254S^ energy‐minimized structure. Residues forming the chol‐binding pocket are depicted in orange and the G254S mutation is highlighted in purple. E) Energy‐minimized structure of IFN‐*γ*R^TM^ mutant. Residues forming the chol‐binding domain are highlighted in orange; L250T, I25T, and G254F mutations are depicted in purple. F) Fluorescence microscopy images of transiently expressed full‐length IFN‐*γ*R2WT‐GFP and IFN‐*γ*R2^TM^‐GFP proteins localization in HAP1^IFN‐*γ*R2KO^ cells (left panel) and PM localization of the wild‐type and mutant receptor (right panel) (*n* = 3 independent experiments). Scale bar = 10 µm. G) In vivo binding of the bifunctional chol probe to full‐length IFN‐*γ*R2WT and IFN‐*γ*R2^TM^ proteins in non‐treated versus IFN‐*γ* stimulated HAP1^IFN‐*γ*R2KO^ cells. Arrow, expected protein size (*n* = 3 independent experiments). H) Quantification of immunoprecipitated radioactivity/protein for chol binding (data are the mean ± SD; *n* = 3 independent experiments). The line on each of the boxes represents the median for that particular data set). Statistical significances were determined with one‐way ANOVA Bonferroni's multiple comparison test (***p < 0.001; **p < 0.01; ns: not significant).

Due to the negative results obtained with the previously described chol‐binding domain, we kept searching for novel putative chol‐binding motifs along the IFN‐*γ*R2TMD sequence that could elucidate how the receptor interacts with chol in living cells. With this purpose, we conducted in silico blind docking experiments between the chol molecule and a minimal energy structure of the IFN‐*γ*R2TMD flanked on both sides by 3 juxtamembrane amino acids that could play an important role in stabilizing this protein–lipid interaction.^[^ ^28^ ^]^ As shown in **Figure** **2**A,B, molecular docking studies showed an optimal fit for chol within a linear amino acid sequence localized in the N‐terminal moiety of the TMD. The putative chol‐binding domain signature QX_2_LIX_2_GX_3_L (highlighted in orange in Figure 2A,B) represents a structural determinant for chol binding. Noticeably, hydrophobic amino acids forming the cavity (L250, I251, G254, and L258) can accommodate and stabilize chol binding by London dispersion forces, and Q247 juxtamembrane amino acid could help to maintain the chol molecule inside the hydrophobic cavity by forming an H‐bond with the chol head group. This domain was found to be highly conserved in the receptor across mammalian species (Figure 2C). To test the chol binding ability of this domain in the IFN‐*γ*R2TMD, we performed site‐directed mutagenesis to replace those residues that might interact directly with chol trying to minimally disrupt TMD features such as ASA, hydrophobicity, or length, which could affect TMD behavior otherwise (Figure S4, Supporting Information). To minimize alterations in the pocket's helical structure properties,^[^ ^29^ ^]^ we reduced cavity hydrophobicity by introducing a Ser residue in place of G254 (IFN*γ*R2^G254S^), highlighted in purple and yellow in Figure 2D and Figure S4B, Supporting Information, respectively. Alternatively, an additional mutant was generated 1) increasing the polarity of the pocket and 2) introducing a bulky aromatic residue to block chol entrance (IFN‐*γ*R2^L250T, I251T, G254F^ triple mutant, from now on IFN‐*γ*R2^TM^) (highlighted in purple in Figure 2E and yellow in Figure S4C, Supporting Information). The TMD mutants showed a similar ASA and hydrophobicity compared to the wild‐type (Figure S4D–G, Supporting Information). To test chol binding in vivo, the full‐length mutants were expressed as GFP fusion proteins (Figure S5A, Supporting Information), showing similar PM localization compared to the WT (Figure S5B, Supporting Information), and interaction experiments were performed as before. Chol signal showed that while IFN‐*γ*R2^G254S^ minimally disrupted binding, IFN‐*γ*R2^TM^ showed a dramatic reduction (>50%) on chol binding as compared to the wild‐type protein, and independently of IFN‐*γ* stimulation (Figure S5C,D, Supporting Information). Identical results were obtained when HAP1^IFN‐*γ*R2KO^ cells not expressing endogenous IFN‐*γ*R2 chain were used (Figure 2F–H). These results support our hypothesis that this newly discovered domain localized within the N‐terminal moiety of the IFN‐*γ*R2TMD is responsible for chol‐binding.

### 2.4 Chol Controls IFN‐*γ*R2 Chain Partition into Lipid Nanodomains

**Figure 3:**
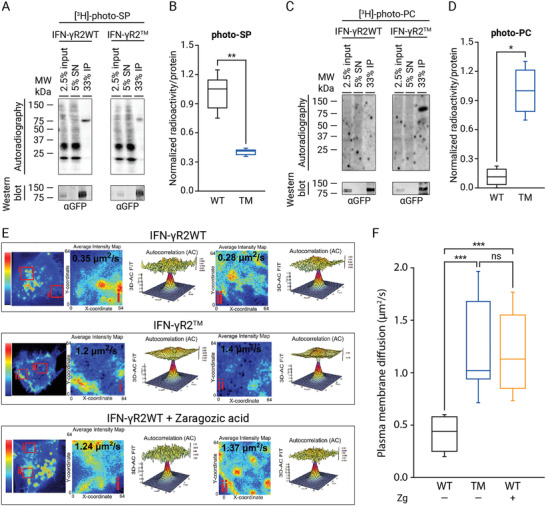
Chol regulates IFN‐*γ*R2 lipid nanodomains partition. A) In vivo photoaffinity binding of SP to IFN‐*γ*R2WT and IFN‐*γ*R2^TM^ in HAP1^IFN‐*γ*R2KO^ cells. B) Quantification of immunoprecipitated radioactivity/protein for SP binding (data are the mean ± SD; *n* = 3 independent experiments). C) In vivo photoaffinity binding of PC to IFN‐*γ*R2WT and IFN‐*γ*R2^TM^ in HAP1^IFN‐*γ*R2KO^ cells. D) Quantification of immunoprecipitated radioactivity/protein for PC binding (data are the mean ± SD; *n* = 3 independent experiments). E,F) Loss of chol‐binding altering the two binding partners independently increases IFN‐*γ*R2 plasma membrane mobility. E) Average intensity projection map (Pseudocolor LUT scale represent normalized average fluorescence intensity) example of transiently expressed IFN‐*γ*R2WT (control or treated with Zg) and IFN‐*γ*R2^TM^ proteins in the cell surface of HAP1^IFN‐*γ*R2KO^ cells (left panels). Close‐up images of subregions corresponding to the two red boxes (i and ii) selected in the left panels in which RICS analysis was performed and displaying the corresponding 2D autocorrelation fit diffusion analysis for selected regions (i and ii); displayed is the 3D representation of the fit with its associated residuals. The resultant IFN‐*γ*R2 diffusion coefficient for each region is marked (right panels). F) RICS quantification of transiently expressed full‐length IFN*γ*R2WT (control or Zg treated) and IFN‐*γ*R2^TM^ protein diffusions through the PM of living HAP1^IFN‐*γ*R2KO^ cells. Data represent the mean of *n* = 3 independent experiments ± SD. *n* = 45 cells/condition. *p*‐values of one‐way ANOVA Bonferroni's multiple comparison test (***p < 0.001; **p < 0.01; *p < 0.05; ns: not significant) are given. The line on each of the boxes represents the median for that particular data set.

What might the function of the direct binding of chol to the IFN‐*γ*R2 protein be? As IFN‐*γ*R activity depends on receptor inclusion into lipid nanodomains,^[^ ^14^ ^]^ we tested whether the loss of chol binding would bring any change to the general lipid environment of the receptor, both in the resting state and after IFN‐*γ* stimulation. Photoaffinity experiments using the bifunctional Sph probe showed that compared to IFN‐*γ*R2WT, the binding of IFN‐*γ*R2^TM^ to SPs in vivo is also highly compromised (>50% of reduction compared to wild‐type) (**Figure** **3**A,B). As expected, when binding of the IFN‐*γ*R2^TM^ to the non‐lipid nanodomains marker (PC) was tested, we observed a tenfold increase in PC labeling over that observed with the IFN‐*γ*R2WT, pointing to a switch of PM partitioning from being included into lipid nanodomains to excluded from them upon loss of chol‐binding (Figure 3C,D). To corroborate this result, a complementary approach was tested, in which HAP1^IFN‐*γ*R2KO^ cells expressing the IFN‐*γ*R2WT chain were treated with zaragozic acid (Zg), an inhibitor of the squalene synthase, the first committed enzyme in mammalian sterol synthesis that diminishes chol levels,^[^ ^30^ ^]^ and binding of the IFN‐*γ*R2WT chain to PC was investigated. As observed in Figure S5E,F, Supporting Information, identical results to the observed with the IFN‐*γ*R2^TM^ were obtained (Figure S5E,F, Supporting Information). However, this IFN‐*γ*R2WT binding to the non‐lipid nanodomain marker PC was not observed when cellular SP levels were reduced using myriocin, a potent inhibitor of serine palmitoyltransferase that catalyzes the first step in sphingosine biosynthesis^[^ ^31^ ^]^ (Figure S5E,F, Supporting Information). We further analyzed changes in IFN‐*γ*R2 surface mobility to study the switch in the lipid environment of the receptor. For this purpose, we investigated receptor diffusion in the PM of HAP1^IFN‐*γ*R2KO^ cells expressing either wild‐type IFN‐*γ*R2WT‐GFP or the IFN‐*γ*R2^TM^‐GFP mutant, using raster imaging correlation spectroscopy (RICS).^[^ ^32^, ^33^ ^]^ RICS is a non‐invasive technique that measures fluorescently labeled proteins' diffusion coefficient on time scales from microseconds to milliseconds in living cells, enabling to chase slow and fast membrane protein movements through the PM at different positions.^[^ ^34^ ^]^ RICS experiments showed that the loss of chol binding leads to a threefold increase in IFN‐*γ*R2^TM^ PM mobility (1.34 µm^2^ s^−1^ ± 0.62) as compared to IFN‐*γ*R2WT (0.39 µm^2^ s^−1^ ± 0.19) (Figure 3E,F), compatible with protein exclusion from the tightly packed lipid nanodomains.^[^ ^33^ ^]^ To ascertain that the increase in IFN‐*γ*R2^TM^ mobility is an immediate effect of the loss of chol‐binding and subsequent changes in lipid environment rather than a direct effect of the introduced mutations in receptor oligomerization, we repeated the same RICS measurements but with a different approach. In this case, we decreased the levels of endogenous chol using Zg and measured the effect of downregulating chol levels on IFN*γ*‐R2WT PM mobility. As observed in Figure 3E,F, identical PM IFN‐*γ*R2WT mobility (1.25 µm^2^ s^−1^ ± 0.51) compared to the mutant version was obtained when endogenous chol levels were decreased. Thus, independently of the binding partner altered (chol or IFN*γ*‐R2), receptor mobility through the PM is affected. Together, these results imply that proper IFN‐*γ*R2 PM targeting and anchoring into lipid nanodomains critically depends on the binding of chol to the receptor.

### 2.5 Chol Stabilizes IFN‐*γ*R Oligomerization

**Figure 4:**
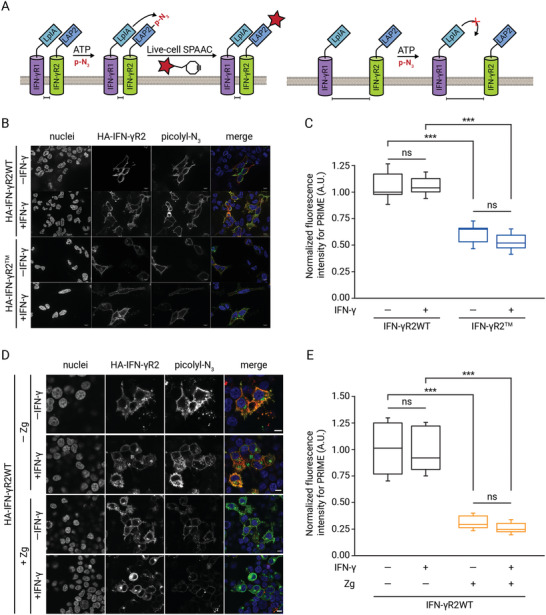
Chol regulates the IFN‐*γ*R oligomeric state in vivo. A) Schematic representation of the ID‐PRIME methodology to visualize IFN‐*γ*R1/IFN‐*γ*R2 specific interaction along the cell surface. The IFN‐*γ*R1 protein is genetically tagged with a mutant of *E. coli* lipoic acid ligase (LpIA) on its extracellular side. IFN‐*γ*R2 is genetically fused with a 13‐amino acids acceptor peptide (LAP2) for LpIA on its extracellular domain. When IFN‐*γ*R1 and IFN‐*γ*R2 proteins interact and are in close proximity, LpIA ligates a picolyl azide (pAz) derivative onto the LAP2 acceptor peptide (left panel). In the case that the two proteins are not in close proximity, pAz transfer does not take place (right panel). In a second step, ligated azide can be detected in living cells by bio‐orthogonal copper‐free click chemistry with a cyclooctyne‐fluorophore conjugated. B) In situ ID‐PRIME detection of IFN‐*γ*R1/IFN‐*γ*R2WT or IFN‐*γ*R1/IFN‐*γ*R2^TM^ interaction in the cell surface of living cells prior to or following IFN‐*γ* stimulation. Cells expressing full‐length Flag‐LpIA‐IFN‐*γ*R1WT and HA‐LAP2‐IFN‐*γ*R2 wild‐type or mutant proteins were labeled with 100 µm pAz + 500 µm ATP for 15 min before and upon IFN‐*γ* stimulation, followed by detection with copper‐free click chemistry, using 20 µm DBCO‐Cy3 for 15 min. Finally, HA‐LAP2‐tagged IFN‐*γ*R2 constructs were visualized by immunofluorescence. Blue, nucleus; green a‐HA; red, pAz. All scale bars are 10 µm. C) Quantification of in situ IFN‐*γ*R1/IFN‐*γ*R2WT and IFN‐*γ*R1/IFN‐*γ*R2^TM^ interaction in cells prior to and upon IFN‐*γ* addition. Data represent the mean of *n* = 3 independent experiments ± SD. *n* = 40 cells/condition. The line on each of the boxes represents the median for that particular data set. D) In situ ID‐PRIME detection of IFN‐*γ*R1/IFN‐*γ*R2WT in the cell surface of living cells previously treated or not with Zg (15 µm) for the last 24 h and handled as described in (B). E) Quantification of in situ IFN‐*γ*R1/IFN‐*γ*R2WT interaction in cells treated or not with Zg (15 µm) before and upon IFN‐*γ* addition. Data represent the mean of *n* = 3 independent experiments ± SD. *n* = 40 cells/condition. Statistical significances were determined with one‐way ANOVA Bonferroni's multiple comparison test (***p < 0.001; **p < 0.01; ns: not significant).

Since the loss of chol binding to IFN‐*γ*R2 subunit changes the lipid environment of the receptor chain and its mobility through the PM, we next investigated whether loss of lipid binding also alters the IFN‐*γ*R1/IFN‐*γ*R2 oligomeric equilibrium in the cell surface of living cells. To assess this possibility, we employed a well‐established in situ protein–protein interaction (PPI) assay named Interaction‐Dependent PRobe Incorporation Mediated by Enzymes (ID‐PRIME).^[^ ^35^, ^36^ ^]^ ID‐PRIME is a versatile two‐step technique for site‐specific labeling of proteins with chemical probes enabling functional studies of PPI in living cells with high spatial and temporal resolution. In the first step, an engineered mutant protein of *E. Coli* origin named LpIA fused to the bait protein A (here IFN‐*γ*R1) catalyzes the covalent tagging of a picolyl Azide (pAz) molecule onto a 13‐amino acid recognition peptide called LAP2 fused to the prey protein B (here IFN‐*γ*R2). In the second step, the covalently tagged pAz molecule is chemoselectively derivatized in vivo with a dibenzylcyclooctyne (DBCO)‐probe via strain promoted alkyne azide cycloaddition. Transfer of the pAZ molecule by the LpIA enzyme can only take place if the two pairs of proteins are in close proximity (**Figure** **4**A left). In the case that the two investigated proteins are not interacting or in close proximity, labeling does not take place (Figure 4A right). First, we tested the feasibility of this methodology to investigate IFN‐*γ*R1/IFN‐*γ*R2 interaction in the surface of living cells (Figure S6A‐E, Supporting Information). Having validated the method, we then applied the ID‐PRIME labeling strategy to cells expressing the bait Flag‐LpIA‐IFN‐*γ*R1WT and the prey HA‐LAP2‐IFN‐*γ*R2WT subunits. We observed prominent labeling of the PM, which suggests a strong PPI between the two subunits (Figure 4B,C). The ID‐PRIME signal at the cell surface was significantly reduced (>50%) when the IFN‐*γ*R2^TM^ mutant was expressed as a prey protein. Similar results were obtained when cells were stimulated with IFN‐*γ* (Figure 4B,C). To ascertain that the cause for the decrease in PPI is a direct effect of lack of chol binding, we repeated the same experiment in cells expressing Flag‐LpIA‐IFN‐*γ*R1WT and HA‐LAP2‐IFN‐*γ*R2WT subunits, but this time downregulating the levels of endogenous chol using Zg. As observed in Figure 4D,E, identical reduction in PPI between the two wild‐type subunits at rest and following IFN‐*γ* activation was observed when chol levels were dysregulated. Thus, independently of the binding partner altered (IFN‐*γ*R2 or chol), receptor oligomerization is affected. Collectively, these results support the notion that chol binding acts as a cofactor regulating IFN‐*γ*R heterodimerization and complex stabilization.

### 2.6 IFN‐*γ*R Signaling Regulation by Chol

**Figure 5:**
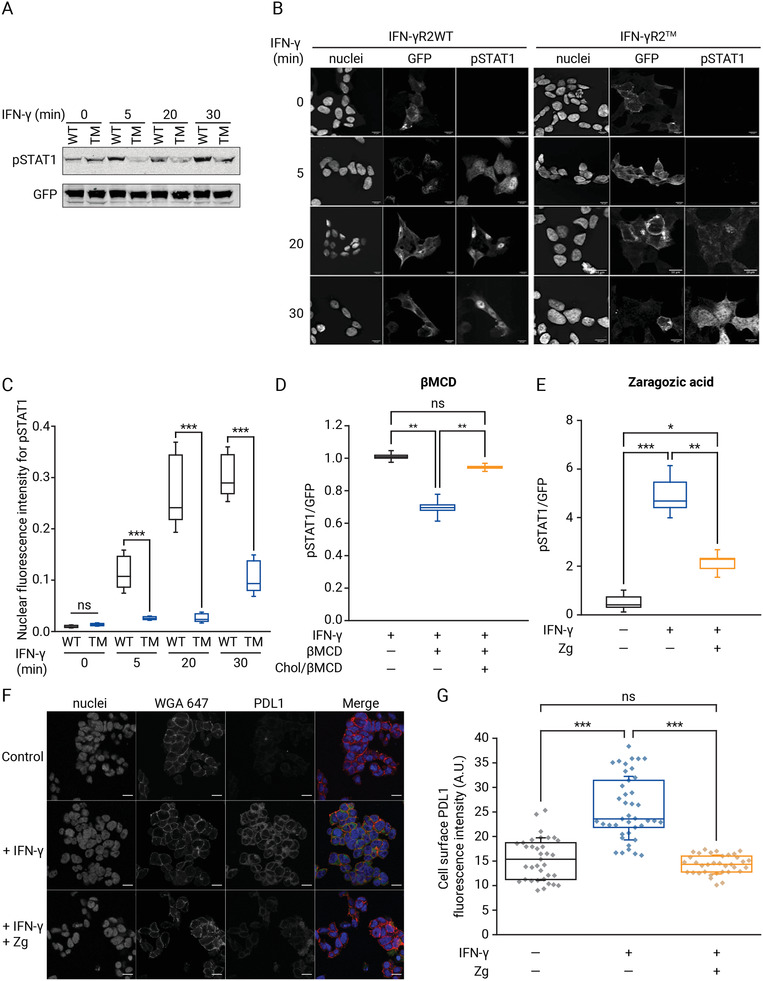
Chol binding regulates IFN‐*γ*R transmembrane signaling. A) Immunoblot for tyrosine phosphorylation of STAT1 (pSTAT1) in HAP1^IFN‐*γ*R2KO^ cells expressing full‐length IFN‐*γ*R2WT or IFN‐*γ*R2^TM^ at different time‐points upon IFN‐*γ* stimulation (representative image from *n* = 3 independent gels). B) Immunofluorescence images of pSTAT1 nuclear translocation in HAP1^IFN‐*γ*R2KO^ cells expressing full‐length IFN‐*γ*R2WT or IFN‐*γ*R2^TM^ proteins at different time points following IFN‐*γ* addition. Nuclei were stained with Hoechst (*n* = 3 independent experiments). Scale bar = 20 µm. C) Quantification of pSTAT1 nuclear translocation in HAP1^IFN‐*γ*R2Ko^ cells expressing full‐length IFN‐*γ*R2WT or IFN‐*γ*R2^TM^ proteins at different time points following IFN‐*γ* stimulation. Data represent the mean of *n* = 3 independent experiments ± SD. *n* = 50 cells/condition. D,E) Chol depletion or synthesis inhibition downregulates STAT1 phosphorylation. D) Quantification of STAT1 phosphorylation in HAP1 cells after 1 h M*β*CD treatment (15 mm), not treated (control) and water‐soluble chol rescue (250 µm, 2 h) followed by 20 min IFN‐*γ* stimulation (*n* = 3 independent experiments). E) Quantification of pSTAT1 in cells after 48 h of Zg (15 µm) treatment followed by IFN‐*γ* stimulation (last 24 h) (*n* = 3 independent experiments). The line on each of the boxes represents the median for that particular data set. F) PD‐L1 cell surface expression in HAP1 cells visualized by immunofluorescence after chol inhibition using 15 µm of Zg (48 h) and IFN‐*γ* stimulation (last 24 h). G) Quantification of PD‐L1 cell surface protein expression in cells treated and handled as described in (E). Data represent the mean of *n* = 3 independent experiments ± SD. n > 36 cells/condition. Statistical significances were determined with one‐way ANOVA Bonferroni's multiple comparison test (***p < 0.001; **p < 0.01; ns: not significant).

A hypothesis arising from the data presented above is that loss of IFN‐*γ*R2 lipid nanodomains targeting and receptor oligomerization would interfere with receptor activation by IFN‐*γ*, and affect IFN‐*γ*R transmembrane signaling activity.^[^ ^14^ ^]^ To test this hypothesis, we analyzed IFN‐*γ* signaling in HAP1^IFN‐*γ*R2KO^ cells expressing either the wild‐type IFN‐*γ*R2 or the IFN‐*γ*R2^TM^ mutant. In cells expressing the IFN‐*γ*R2WT subunit, IFN‐*γ* stimulation led to efficient activation of the JAK/STAT signaling pathway (**Figure** **5**A). As expected, STAT1 activation resulted in protein phosphorylation and nuclear translocation (Figure S7A, Supporting Information). In contrast, cells expressing the IFN‐*γ*R2^TM^ showed a significant decrease in JAK/STAT signaling activation upon IFN‐*γ* stimulation (Figure 5A and Figure S7A, Supporting Information). As a result, STAT1 phosphorylation and nuclear translocation were drastically inhibited (>50% as compared to IFN‐*γ*R2WT) (Figure S7B, Supporting Information). Next, we investigated whether the inhibition of STAT1 phosphorylation and nuclear translocation triggered by the lack of INF‐*γ*R2/chol interaction is caused by a delay of JAK/STAT activation upon cytokine stimulation. Cells expressing the IFN‐*γ*R2WT subunit showed a fast JAK/STAT activation. After 5 min of IFN‐*γ* stimulation, STAT1 activation and nuclear translocation was observed (Figure 5B,C). However, in the presence of the IFN‐*γ*R2^TM^, a noticeable delay on JAK/STAT signaling activation occurred. Only after 15 min of continuous IFN‐*γ* stimulation, STAT1 phosphorylation starts to be appreciated (Figure 5B,C). To further support the role of chol as the molecular determinant of IFN‐*γ*R signaling, we treated wild‐type HAP1 cells with methyl‐*β*‐cyclodextrin (M*β*CD), which depletes chol from cellular membranes. As observed in Figure 5D and Figure S7C, Supporting Information, depletion of chol leads to a marked decrease in STAT1 phosphorylation. Interestingly, when the M*β*CD effect was reversed by reloading cells with water‐soluble chol, normal IFN‐*γ*R transmembrane signaling was restored (Figure 5D and Figure S7C, Supporting Information). Admittedly, results obtained with M*β*CD can be of limited physiological relevance since this chol removal agent has pleiotropic effects in the cell.^[^ ^6^, ^7^ ^]^ Thus, to confirm the chol removal results obtained, STAT1 phosphorylation assays were repeated in the presence of the chol synthesis inhibitor Zg, which has less biological side effects. Identical STAT1 phosphorylation impairment was observed in the presence of Zg (Figure 5E and Figure S7D, Supporting Information). Our results altering the two binding partners independently (chol or IFN‐*γ*R2) demonstrate that the binding of chol to the IFN‐*γ*R2 subunit is required for correct IFN‐*γ*R transmembrane signaling.

Finally, it is well known that the IFN‐*γ*/JAK/STAT1 signaling axis regulates the inducible expression of a vast array of proteins involved in antiviral, antigen, or pro‐tumoral functions.^[^ ^37^ ^]^ Among others, the inducible expression of the immune checkpoint PD‐L1 protein in the cell surface of cancer cells has been described to be primarily coordinated by the IFN‐*γ* signaling pathway.^[^ ^17^, ^38^ ^]^ PD‐L1 binds to its receptor PD‐1 present in T cells and inhibits the antitumor response, a process known as adaptive immune resistance, which ultimately leads to immune evasion.^[^ ^17^, ^18^, ^39^ ^]^ A hypothesis that emerges from this work is that blocking the IFN‐*γ* signaling cascade by altering the levels of chol could have a direct impact on PD‐L1 cell surface protein expression in cancer cells, which was studied as a representative effect of IFN‐*γ* signaling cascade disruption. To test this, HAP1 cells previously treated with the chol synthesis inhibitor (Zg) were subjected to IFN‐*γ* exposure, and PD‐L1 cell surface levels were visualized and quantified. Disrupting the IFN‐*γ* signaling axis by downregulating chol levels markedly decreases cell surface expression of PD‐L1 to levels similar to the observed in not stimulated cells (Figure 5F,G). To investigate if the observed decrease of PD‐L1 cell surface expression is a general mechanism taking place in different cancer types, we screened two additional cancer cell lines, HeLa and the highly aggressive and invasive MDA‐MB‐231. We found that disrupting the IFN‐*γ*/IFN‐*γ*R/chol signaling axis is a general mechanism to control PD‐L1 cell surface protein expression in all tested cancer cells (Figure S7E‐H, Supporting Information).

### 2.7 A General Chol‐Binding Motif in Mammalian Membrane Proteins

**Figure 6:**
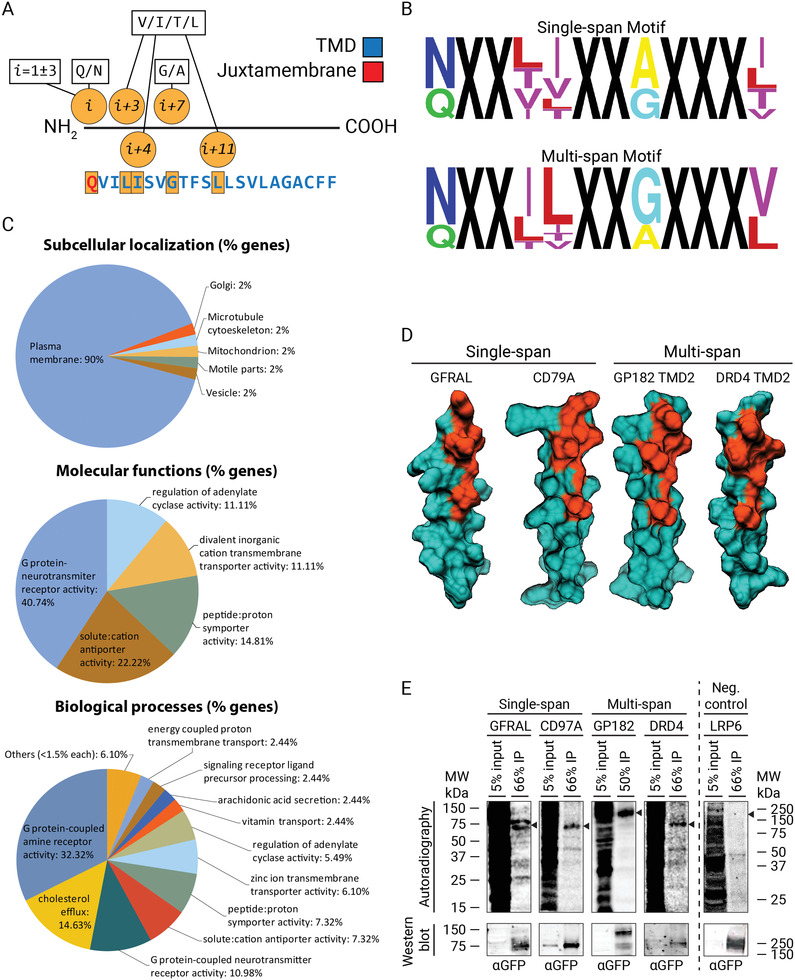
A general chol‐binding motif in mammalian membrane proteins. A) Relaxed motif signature used for bioinformatic studies using MOPRO for screening potential chol‐binding single‐ and multi‐span protein candidates in mammalian membrane proteome data sets. B) TMDs motifs significantly overrepresented with a *p*‐value ≤ 0.05 and *z*‐score > 2.5 were used to generate a single‐ (top panel) and multi‐span (bottom panel) sequence logo, where the letter size reflects the probability to find this amino acid at that particular position. C) Bioinformatic studies showing the subcellular distribution, molecular, and biological functions associated with the different putative chol‐binding membrane protein candidates identified in (A). D) 3D Minimal‐energy structures of the four candidates tested for chol‐binding in living cells. The IFN‐*γ*R2 like chol‐binding domain is highlighted in orange, whereas the rest of the protein is depicted in dark green. E) In vivo labeling of GFP‐tagged constructs of candidates GFRAL, CD79A, GP182, DRD4, and the negative control LRP6. HAP1 cells were labeled with 100 *μ*Ci bifunctional Chol (3 µm) for 6 h, UV‐irradiated, lysed, and subjected to IP using an antibody against GFP. Corresponding radioactivity recovered for each protein candidate was detected and visualized by autoradiography and western blot analysis, respectively. Arrow, expected protein sizes (*n* = 3 independent experiments).

To determine if the chol‐binding motif identified in the IFN*γ*‐R2 TMD is a general motif present in other proteins, we generated a “relaxed” motif to search for other chol‐binding membrane protein candidates. We allowed different permutations of the *β*‐branched amino acid Val and the non‐branched amino acids Leu and Gly in the lipid‐binding crevice and the polar uncharged amino acid Gln in the interface position: (Q/N)XX(V/I/T/L)(V/I/T/L)XX(G/A)XXX(V/I/T/L) (**Figure** **6**A). This new relaxed motif corresponds to 256 different possible motifs (2 × 4 × 4 × 2 × 4), generating a vast number of candidate proteins if directly used to screen an extensive set of mammalian proteins. To overcome this issue, we used a simple motif‐probability algorithm named MOtif PRObability (MOPRO) that identifies membrane proteins containing the putative chol‐binding motif in a database containing proteomes from mammalian organisms (see Experimental Section for further details).^[^ ^40^ ^]^ We applied MOPRO to search for putative chol‐binding candidates in single‐span membrane proteins in a homology‐reduced mammalian protein dataset, and we identified eight over‐represented chol‐binding motifs with a stringent *p*‐value ≤ 0.05 and *z*‐score > 2.5 (Table S1, Supporting Information) that fulfill the “relaxed” motif defined in Figure 6A. These over‐represented motifs were then used to search for candidates, this time in a non‐homology reduced‐mammalian dataset. In this way, a total of 12 novel single‐span membrane proteins were found (Table S2, Supporting Information). Next, we tested whether IFN‐*γ*R2‐like chol‐binding domains are not only restricted to single‐span membrane proteins but are also found in multispan membrane proteins. To this end, we repeated the same procedure described above and identified ten over‐represented motifs (Table S1, Supporting Information) and 38 novel chol‐binding proteins (Table S2, Supporting Information) in a dataset comprising all predicted transmembrane spanning membrane proteins in complete proteomes from mammalian organisms. Motifs with a *p*‐value ≤ 0.05 and *z*‐score > 2.5 were used to generate a sequence logo for single‐ and multi‐span membrane proteins, with the letter size corresponding to the probability of finding this amino acid at that position (Figure 6B). The novel chol‐binding single‐ and multi‐span membrane protein candidates are predominantly localized at the PM (Figure 6C). This is consistent with the fact that the PM is the highest chol‐enriched organelle in a cell.^[^ ^4^ ^]^ Strikingly, a high number of chol‐binding protein candidates are GPCRs and ion channels. The identified candidate proteins are involved in a wide range of molecular and biological processes, including G protein‐coupled amine receptor, G‐protein neurotransmitter receptor activity, chol efflux, solute:cation antiporter activity, or regulation of adenylate cyclase activity, among others (Figure 6C). To validate the list of chol‐binding protein candidates, four recombinant candidates—two single‐span membrane proteins: the GDNF family receptor alpha‐like (GFRAL) and the B‐cell antigen receptor complex‐associated protein alpha chain (CD79A); and two multispan membrane protein: the G‐protein coupled receptor 182 (GPR182) and the D(4) dopamine receptor (DRD4) (Figure 6D)—were transiently expressed as GFP‐tagged fusion proteins in HAP1 cells and tested for interaction with chol in vivo. Low‐density lipoprotein receptor‐related protein 6 (LRP6) was selected as a negative control since it does not contain the putative chol‐binding signature and localizes to the PM facing the bulk pool of chol. Immunofluorescence analysis of cells expressing the different GFP‐tagged candidate proteins showed that all the candidates localize mainly at the PM (Figure S8, Supporting Information). To probe for chol‐binding, cells were fed with the bifunctional chol probe, followed by UV‐irradiation, immunoprecipitation, western blotting, and autoradiography as described before. Strikingly, all the four candidates exhibit a high extent of chol interaction in vivo, whereas no interaction was observed with LRP6, a protein not containing the chol‐binding motif (Figure 6E). The specific interaction of all single‐ and multi‐span membrane proteins tested identified in the bioinformatic analysis suggest that many other protein candidates identified in this study could potentially bind chol in vivo, and validate the new chol‐binding domain as a general motif in the mammalian membrane proteome.

## 3 Discussion

Our data presented here uncovered a hitherto unprecedented interaction between chol and the TMD of a cell surface signaling receptor implicated in the coordination of innate and adaptive immunity. By combining in silico predictions with molecular and chemical cell biology experiments, we have identified the structural signature within the membrane receptor required for chol binding. Compared to other previously identified chol‐binding domains found in X‐ray structures, the newly identified chol‐binding motif (QX_2_LIX_2_GX_3_L) shares general structural features present in many crystal protein structures.^[^ ^28^ ^]^ First, chol molecule positioning into the cavity is described to be stabilized by hydrogen bonding between the sterol 3*β*‐hydroxyl group and the polar side chain of a Gln or Asp residues. Second, as observed for the IFN‐*γ*R2 chol‐binding motif, Ile, Val, and Leu are commonly found partners of the hydrophobic tetracyclic part of the chol molecule. The presence of these partners is not surprising, considering that the three amino acids have high hydropathy indexes.^[^ ^41^ ^]^ Finally, the chol isoctyl chain has previously been found, like in this work, to be stabilized by *β*‐branched amino acids or Leu residues. It is important to mention that docking experiments only provide a putative snapshot of how chol could fit into the binding pocket and do not necessarily reflect the native structural conformation. Future biochemical and structural experiments will help elucidate how chol molecule sits within the IFN‐*γ*R2‐TMD in complex systems. However, the technical repertoire of biochemical in vitro methods to study intramembrane protein–lipid interactions is limited and obtaining X‐ray structures of membrane proteins in their native environment is still challenging. Alternatively, in silico molecular dynamic simulations could give important information at the atomistic level and provide a structural view of how chol could fit into the binding domain in more complex biological systems.

**Figure 7:**
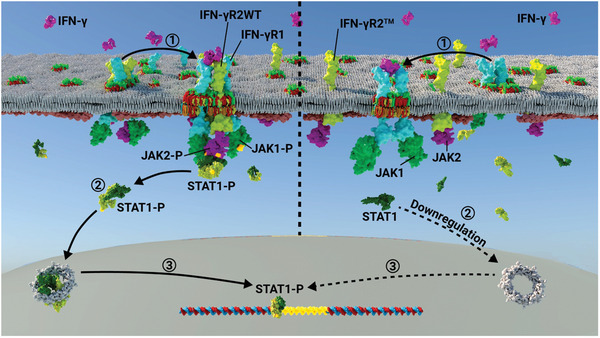
Model of IFN‐*γ*R transmembrane signaling regulation by chol. Left: 1) chol binding to the IFN‐*γ*R2‐TMD targets and maintains the receptor chain into lipid nanodomains and stabilizes IFN‐*γ*R heterodimerization. Binding of an IFN‐*γ* dimer to the IFN‐*γ*R1 and IFN‐*γ*R2 subunits undergoes receptor intracellular conformational changes leading to transactivation of IFN‐*γ*R bound JAK1 and JAK2 proteins and their phosphorylation (JAK1‐P and JAK2‐P). This activated complex serves as a docking site for cytosolic STAT1 complex binding and phosphorylation (STAT‐1P). 2) STAT1‐P translocates to the nucleus as a transcription factor and 3) binds to IFN‐*γ* activate site elements (GAS) present in the promoter of some IFN‐stimulated genes (ISGs), thereby controlling their transcription. Right: 1) disruption of chol‐binding in the IFN‐*γ*R2 chain decreases receptor insertion into lipid nanodomains and increases PM IFN‐*γ*R2 diffusion, thereby downregulating receptor activation by IFN‐*γ* dimers, 2) STAT‐1P nuclei translocation, and 3) ISGs transcription.

The results described in this work reveal a novel and unexpected molecular mechanism in which the binding of chol regulates 1) receptor PM lateral segregation, 2) IFN‐*γ*R oligomerization, and 3) downstream transmembrane signaling. Our data favor a model in which chol plays a critical role by targeting and maintaining the IFN‐*γ*R2 chain into pre‐existing lipid nanodomains stabilizing IFN‐*γ*R1/IFN‐*γ*R2 receptor heterodimerization and enabling optimal activation of the JAK/STAT signaling cascade (**Figure** **7**, left). Conversely, disruption of the IFN‐*γ*R2TMD–chol interaction affects receptor segregation and retention into lipid nanodomains. The resulting increase in receptor PM mobility drastically affects the IFN‐*γ*/IFN‐*γ*R/JAK/STAT1 signaling axis (Figure 7, right). Alternatively, binding of chol to the IFN‐*γ*R2TMD could act as a nucleation site for the generation of new specific lipid nanodomains required for correct receptor transmembrane signaling activation. Regarding the mechanism behind targeting to lipid nanodomains, different alternatives to membrane protein recruitment have been introduced in the past.^[^ ^42^ ^]^ Among others, palmitoylation, TMD length, amino acid composition, and ASA are described as structural determinants for protein affinity to lipid nanodomains.^[^ ^9^ ^]^ However, for membrane proteins not filling such molecular features, nature could exploit different mechanisms like the one described in this work, based on specific protein–lipid interactions to target and maintain a membrane protein into lipid nanodomains. These different mechanisms could act alone or in combination to control and maintain spatiotemporal receptor lateral segregation in the cell surface of living cells. A question that remains to be addressed is how the IFN‐*γ*R1 chain is laterally segregated and maintained into lipid nanodomains. In this context, we have previously described an SP binding domain localized within the IFN‐*γ*R1TMD that could play a similar role to that carried out by chol in this work.^[^ ^12^ ^]^ It is important to mention that our experiments in IFN‐*γ*R1/IFN‐*γ*R2 interaction highlight the role of chol as an important cofactor stabilizing IFN‐*γ*R heterodimerization, but cannot distinguish between the two receptor heterodimerization models that have been previously proposed: 1) triggered by ligand binding or 2) ligand‐induced conformational changes of a pre‐assembled IFN‐*γ*R complex.^[^ ^16^, ^43^ ^]^ The control of the IFN‐*γ*R activity by chol shown in this work is important beyond the cell signaling scenario and provides a mechanistic explanation that could help to understand how the binding of specific lipids can modulate membrane protein activities in general. Eventually, these lipid–protein interactions will help us elucidate why biological membranes are built up with such astonishing lipid diversity.^[^ ^4^, ^8^ ^]^ The molecular mechanism unraveled here is of particular interest in many human diseases where IFN‐*γ* signaling plays a central role, such as atherosclerosis, pathogen infection, and cancer.^[^ ^19^, ^20^ ^]^ Specifically, our findings shed light to the plausible molecular mechanism evolved by many pathogens to escape from the immune response by depleting chol from the cell membrane of infected cells and shutting down the IFN‐*γ* signaling cascade, which in turn blocks the immune response and ultimately leads to immune evasion.^[^ ^44^ ^]^ Moreover, the inhibition of PD‐L1 cell surface protein expression caused by blocking the IFN‐*γ* signaling cascade derived from downregulating chol levels in cancer cell lines opens up a new route for the development of a novel strategy that, alone or in combination with immunotherapy, can help to better act against cancer.^[^ ^17^, ^18^ ^]^ Indeed, a recent study showed that statin treatment decreases immune checkpoints expression in the cell surface of immune cells.^[^ ^45^ ^]^ However, it is important to stress that unlike squalene synthase inhibitors like Zg that block chol synthesis predominantly, statins inhibit the hydroxymethylglutaryl‐coenzyme A (HMG‐CoA) reductase enzyme and mevalonate formation. Hence, its inhibition affects the production of chol, isoprenoids, and ubiquinone, among others, showing a broader range of pleiotropic cellular effects, making the association between statins outcome and chol lowering exclusively challenging.^[^ ^46^ ^]^ Finally, bioinformatic analysis predict that the novel chol‐binding domain described in this work is a general motif present in a large number of single‐ and multiple‐span membrane proteins. These bioinformatic studies can be used as a starting point for functional studies of individual membrane protein candidates. In this sense, the single‐membrane protein candidate CD79A tested in this study for in vivo chol binding has been described to localize into lipid nanodomains to carry out their signaling functions.^[^ ^47^ ^]^ Thus, it is tempting to speculate that CD79A, upon ligand activation, may require chol binding to direct and maintain the receptor into specified domains and additionally or alternatively regulate receptor heterodimerization and cell signaling cascade. Besides, a large number of multispan membrane proteins containing the IFN‐*γ*R2‐like chol‐binding domains correspond to ion channels and GPCR proteins. Notably, the new chol‐binding motifs in GPCR candidates are over‐represented in TMD2. Previously, it has been demonstrated that GPCR receptor TMD2 participates in the formation of the receptor's binding pocket. In the resting state, a highly conserved Asp residue within the TMD2 faces the binding pocket, and upon activation through a pivoting movement, TMD2 pushes away the Asp residue that undergoes an intermolecular bonding network between TMD2 and TMD7, stabilizing the active form of the receptor.^[^ ^48^ ^]^ In this TMD rotational process, chol‐binding to TMD2 could initiate TMD2 pivoting movement and stabilize TMD2‐TMD7 network interactions.

In summary, our results have deciphered the role that chol plays in modulating cell surface IFN‐*γ*R functions and identified the IFN‐*γ*R2TMD–chol interaction as a potential drug target for the regulation of the IFN‐*γ*/IFN‐*γ*R1/IFN‐*γ*R2/chol signaling axis in a large number of diseases where IFN‐*γ* signaling is intimately involved.

## 4 Experimental Section

### Antibodies and Reagents

The following antibodies were purchased from Abcam (Cambridge, CA): rabbit polyclonal antibodies against GFP, HA, and PD‐L1 (Alexa Fluor 488), a monoclonal antibody against FLAG. Rabbit polyclonal anti‐pSTAT (Tyr701) was obtained from BD Bioscience (San Jose, CA). For immunofluorescence staining, Alexa Fluor‐488, ‐546, and ‐647 goat anti‐rabbit or anti‐mouse secondary antibodies (Thermo Fisher Scientific, Waltham, MA) were used. For western blot detection, IRDye 700CW and IRDye 800CW anti‐rabbit or anti‐mouse secondary antibodies (LI‐COR Bioscience, Lincoln, NE) were used. Recombinant human IFN‐*γ* and myoricin were purchased from Merck (Darmstadt, Germany). Sulfo‐Cy3‐DBCO was obtained from click chemistry tools (Scottsdale, AZ). ATP disodium hydrate salt and acetate tetrahydrate were purchased from Sigma Aldrich (Saint Louis, MO). Zaragozic acid A trisodium salt was purchased from Sta. Cruz Biotechnology (Dallas, TX). M*β*CD was obtained from Sigma Aldrich (San Luis, MO). For transfection FugeneHD from Promega (Madison, WI) and Opti‐MEM from Thermo Fisher Scientific (Waltham, MA) were used. For immunoprecipitation Protein A Sepharose Fast Flow Beads were purchased from GE Healthcare (Chicago, IL).

### Cell Lines

Chinese hamster ovary cells (CHO), human embryonic kidney cells (HEK293), MDA‐MB231 breast cancer cells, and HeLa adenocarcinoma cells were obtained from ATCC (Manassas, VA) and maintained in modified Eagle's medium alpha or Dulbecco's Modified Eagle Medium (DMEM) supplemented with Glutamax, 10% fetal calf serum (FCS), 100 U mL^−1^ penicillin, 100 U mL^−1^ streptomycin and 2 mm L‐glutamine at 5% CO_2_ and 37 °C. HAP1WT and HAP1^IFN‐*γ*R2KO^ cells were purchased from Horizon Discovery (Waterbeach, CA) and maintained in IMDM medium supplemented with 10% FCS, 100 U mL^−1^ penicillin–streptomycin at 5% CO_2_ and 37 °C. Cells were monthly tested for mycoplasma by PCR.

### Plasmids

IFN‐*γ*R1 and IFN‐*γ*R2 cDNAs were cloned into peGFP‐N1 and phRLucN3 (Clontech, Mountain View, USA) as previously described.^[^ ^14^ ^]^ Synthetic Flag‐LpIA‐IFN‐*γ*R1 and HA‐LAP2‐IFN‐*γ*R2 constructs were generated (Genscript, Piscataway, NJ) and flanked with NheI and EcoRI restriction sites. Site directed mutagenesis was performed by Genscript (Piscataway, NJ). GPR182, CD79A, GFRAL, and DRD4 cDNAs were cloned into pcDNa3.1(+)‐C‐GFP (Genscript, Piscataway, NJ). pYFJ16‐LpIA (W37V) for *E. coli* expression was a gift from Alice Ting (Addgene plasmid #34838, http://n2t.net/addgene:34838; RRID: Addgene_34838). Cav1‐GFP was a gift from Ari Helenius (Addgene plasmid # 14433; http://n2t.net/addgene:14433; RRID:Addgene_14433)‐LRP6‐GFP construct was a gift from Sergio Perez Acebron (Centre for Organismal Studies, Heidelberg, Germany)

### Synthesis of Compounds

Photoactivatable and radiolabeled cholesterol (^3^[H]‐photo‐chol), sphingosine (^3^[H]‐D‐*erythro*‐photoSph), and 10‐azi‐stearic acid (10‐ASA) were synthesized as described in ref. [12]. Synthesis of pAz molecule was carried out as described in ref. [36].

### In Vivo Photoaffinity Labeling

Cells were transfected using FuGENE HD as a transfection reagent according to the instructions of the manufacturer (Promega). A total of 5 µg of cDNA and 15 µL of FuGENE HD in 200 µL of Opti‐MEM (Invitrogen) were used to transiently transfect a subconfluent 10 cm dish of cultured cells. For photoaffinity labeling experiments, cells expressing the protein of interest (IFN‐*γ*R1 and IFN‐*γ*R2 variants, and chol interaction motif candidates) were labeled with the different photoactivatable precursors (^3^[H]‐photo‐chol, ^3^[H]‐photo‐SP, or ^3^[H]‐choline + 10‐ASA) as described in refs. [22, 23]. For competition experiments, photoactivatable lipids were administrated together with increasing amounts of native chol or sph (precursor of SP) to the cells. Briefly, cells were washed with PBS, and incubated with lipids using the following conditions: 3 µm of an ethanolic solution of ^3^[H]‐photo‐chol (100 µCi), 2 µm of an ethanolic solution of ^3^[H]‐photo‐Sph (60 μCI), or ^3^[H]‐choline (50 *μ*Ci) combined with 10‐ASA (100 µm) was mixed with 10 mL of culture medium with 10% delipidated FCS. After labeling, the medium was removed, and the cells were washed twice with PBS and stimulated or not with IFN‐*γ* for 5 min. After stimulation, cells were washed twice with PBS, and all subsequent steps were performed at 4 °C. Cells were UV‐irradiated (Sylvania R 100 W) in 5 mL of PBS for 5 min on ice. After irradiation, PBS was removed, and the cells were harvested in 0.8 mL of PBS by scraping. Cells were pelleted (16 000 × *g*, 5 min) and lysed in lysis buffer (50 mm HEPES‐NaOH, pH 7.4, 100 mm NaCl, 5 mm EDTA, 1% Triton X‐100 [v/v], 0.5% deoxycholate [w/v], and protease inhibitor cocktail) for 1 h. After lysis, nuclei were removed by centrifugation (3000 × *g*, 10 min) and the supernatant (100 µL) was subjected to immunoprecipitation using protein A sepharose beads and anti‐GFP antibody. After overnight incubation, immune complexes were washed with lysis buffer (five times, 1 mL) and proteins were eluted using SDS‐PAGE sample buffer. Samples were subjected to SDS‐PAGE (10–20% Tris/Tricine gradient gel, Invitrogen) and western blotting. Radioactively labeled proteins were detected by digital autoradiography (*β*‐Imager 2000, Biospace). Normalized radioactivity/protein was calculated from the signal density obtained from autoradiography and corresponding western blots.

### Confocal Microscopy

Cells were grown on glass coverslips, and after 24 h GFP‐tagged construct transfection cells were washed three times with PBS and fixed with 4% PFA for 15 min at RT. Next, cells were rinsed three times with PBS, coverslip mounted, and immediately visualized by fluorescence microscopy. For immunofluorescence assays, after fixation, cells were rinsed three times with PBS and permeabilized with 0.1% Triton X‐100 in PBS for 10 min at RT. Cells were washed three times and incubated in blocking buffer (1% BSA, PBS pH 7.4) for 30 min to avoid unspecific antibodies binding. After blocking, cells were incubated with the primary antibody in 1% BSA, PBS pH 7.4 for 1 h. Finally, cells were rinsed three times with PBS and incubated with the secondary antibody in 1% BSA, PBS pH 7.4 for 30 min, washed three times with PBS, and coverslip mounted. All imaged were acquired with a Zeiss Apotome microscope equipped with an argon laser beam. Image processing was performed using FIJI‐ImageJ software.

### IFN‐*γ*R2 Variants Plasma Membrane Localization

CHO and HAP1^IFN‐*γ*R2KO^ cells were grown on glass coverslips, and transfected with GFP‐tagged IFN‐*γ*R2WT or IFN‐*γ*R2^TM^ constructs. 24 h after transfection cells were washed three times with PBS and incubated for 10 min at 37 °C with 5 µg mL^−1^ WGA‐Alexa Fluor 647 in PBS. After labeling, cells were washed three times with PBS, and fixed with 4% PFA for 15 min at RT. After three washes with PBS, cells were coverslips mounted. IFN‐*γ*R2WT‐GFP and IFN‐*γ*R2^TM^‐GFP cell surface expression was quantified with FIJI‐ImageJ software by calculating the GFP PM signal, using the WGA‐Alexa Fluor 647 staining to create a PM mask.

### Protein Candidate's Subcellular Localization

HAP1 cells were grown in a six well‐plate and transfected with GFP‐tagger chol binding protein candidate constructs for 24 h. After transfection, cells were washed three times with PBS and detached with cell dissociation buffer. PM staining was performed in suspension with WGA‐Alexa Fluor 647 diluted in PBS (5 µg mL^−1^) for 10 min at room temperature. Next, cells were washed to remove non‐reacted probe and fixed with 4% PFA for 15 min. Finally, cells were attached to a coverslip using a Cellspin system mounted and visualized by confocal microscopy.

### STAT1 Activation and Nuclear Translocation

HAP1^IFN‐*γ*R2KO^ cells expressing IFN‐*γ*R2WT or IFN‐*γ*R2^TM^ were treated with or without 1000 U mL^−1^ of IFN‐*γ* at 37 °C for the indicated times. For biochemical analysis, cells were washed with ice‐cold PBS and lysed in lysis buffer (50 mm HEPES‐NaOH pH 7.4, 100 mm NaCl, 5 mm EDTA, 1% Triton X‐100 [v/v], 0.5% deoxycholate [w/v], protease inhibitor cocktail, and phosphatase inhibitor) for 1 h. After lysis, nuclei were removed by centrifugation (3000 × *g*, 10 min), and 50% of lysate was analyzed by SDS‐PAGE and western blot analysis using a LI‐COR Odyssey CLx Imaging System. For pSTAT1 nuclear translocation immunofluorescence analysis, cells were grown on coverslips and transfected with IFN‐*γ*R2WT‐ or IFN‐*γ*R2^TM^‐GFP tagged constructs using FuGENE HD, treated with IFN‐*γ* as described above, and then fixed with cold methanol for 15 min at −20 °C. After washing with PBS three times, cells were subjected to a second round of permeabilization using 0.4% Triton X‐100 for 15 min. After permeabilization, cells were rinsed with PBS, incubated in blocking buffer for 30 min, and incubated with primary antibody anti‐pSTAT1 for 1 h at room temperature. Finally, cells were rinsed three times with PBS and incubated with the secondary antibody in 1% BSA, PBS pH 7.4 for 30 min, and coverslips mounted in media containing DAPI. pSTAT1 nuclear translocation was quantified with FIJI‐ImageJ software by calculating the pSTAT1 nuclear signal (nuclei mask was realized with DAPI staining).

### Docking Experiments

3D energy‐minimized structure of the TMDs was obtained using the Biochemical Algorithms Library (BALL) viewer 1.4.2 software employing the MMFF94 force field.^[^ ^49^ ^]^ The solvent excluded (van der Waals) surfaces were simulated, and the structure was set to a maximum energy difference of 0.0001 kJ mol^−1^ (15 000 interactions). Cholesterol structure was obtained from the PubChem Compound Database (PubChem CID: 5997). Docking experiments between the acceptor transmembrane receptor and the cholesterol ligand were performed using the AutoDock Vina software (Scripps Research Institute),^[^ ^50^ ^]^ and binding energies were calculated. The ligand and the target transmembrane protein were prepared following the standard procedure of ligand and protein preparation. Finally, the prepared files were submitted to AutoDock Vina. For blind docking experiments, the grid box covers the complete proteins, whereas, for biased assays, the grid box wraps only the target region in the protein. The docked complexes were analyzed using Discovery Studio 3.1 visualizer or Chimera UCS software. Besides, blind protein–protein docking experiments were performed using the SwarmDock software (Francis Crick Institute).^[^ ^51^ ^]^ The lowest 3D energy structure of the TMDs was obtained as described above.

### Bioinformatical Analysis

The amino acid sequence of IFN‐*γ*R2 was obtained in FASTA format from UniProt. A search for chol‐binding domain CRAC and CRAC‐like motifs was performed using EMBROSS fuzzpro program, sequences given as a motif search pattern were: [LV]‐X_(1–5)_‐Y‐X_(1–5)_‐RK], [LV]‐X_(1–5)_‐F‐X_(1–5)_‐[RK], and [RK]‐X_(1–5)_‐F‐X_(1–5)_‐[LV].

### Protein Sequence Alignment

All IFN‐*γ*R2 protein sequences from different mammalian species were downloaded in FASTA format from Uniprot.^[^ ^52^ ^]^ Multiple sequence alignment was performed using the ClustalW program.

### Motif Probability Analysis

MOPRO was a tool that allows to test if the chol‐binding signature found within the IFN‐*γ*R2‐TMD was unique to this membrane protein, or rather, represented a general motif found in other proteins in mammalian membranes proteomes. MOPRO analysis for single‐ and multi‐span membrane proteins was performed as described in ref. [40]. Briefly, in a first step, MOPRO removed motifs not generating a statistically significant overrepresentation of hits over a homology reduced sequence data set. This was done by randomizing each TMD sequence of the data set using a swap algorithm. The algorithm swapped the position of two randomly selected residues for each TMD and the number of swaps was set equal to the number of residues for each TMD. Through this process, the algorithm ensured that on average, each amino acid had swapped position two times. Then, the number of occurrences of motif "i" in the randomized TMD data set, Ri, was obtained. Next, the randomization procedure was repeated 10 000 times to obtain the exact Ri value distribution expected for motif "i" over a TMD data set with randomized sequences. From this distribution, the *p*‐value and *z*‐score for motif "i" were calculated. Finally, the motifs that were found to be over‐represented were used to screen for chol‐binding protein candidates. Candidates were acquired from a 90% homology reduced mammalian data set containing 9981 proteins. This homology reduction was performed using cd‐hit.^[^ ^40^, ^53^ ^]^ The 8 and 12 novel motifs for single‐ and multi‐span membrane proteins, respectively, were used. TMDs were predicted using SCAMPI by extending the predicted TMDs by three amino acids upstream and downstream.

### Raster Imaging Correlation Spectroscopy

HAP1^IFN‐*γ*R2KO^ cells were seeded onto imaging dishes (Ibidi 81156) in IMDM media and transfected with GFP‐tagged IFN‐*γ*R2WT or IFN‐*γ*R2^TM^. RICS acquisitions were recorded on a commercial LEICA SP8 3X STED SMD confocal microscope (Leica Microsystems, Manheim, Germany), with an HCX PL APO 63x/1.2NA CORR CS2 water immersion objective and using a WLL as pulsed laser source. Relative power, as it appears in the LAXs software, was always below 2%. The images shown were representative of the experiments used for quantification out of a larger set of time lapses that were also analyzed for statistical purposes. Diffusion analysis by RICS was examined using SimFCS 4 software (G‐SOFT Inc.), as previously described in ref. [32]. Point spread function was determined as described elsewhere.^[^ ^32^ ^]^ RICS images series (256 × 256 pixels) were taken using either an 8 or 4 µs dwell time with no difference in the diffusion yielded between them. Each time‐lapse was taken for 200–300 total frames. From each full‐frame time‐lapse, a smaller region of interest was selected (32 × 32 pixels), and the diffusion coefficient was obtained by fitting the experimental 2D autocorrelation function to a single diffusion mode. The 2D autocorrelation map was then fitted to obtain a surface map, employing the characterized waist value and the appropriate acquisition values for line time and pixel time.

### Cholesterol Synthesis Inhibition

The impact of cholesterol synthesis inhibition on IFN‐*γ* signaling was studied by treatment of HAP1 cells seeded in 35 mm dishes with the sterol synthesis inhibitor Zg. Cells were incubated with 15 µm of Zg for 48 h followed by IFN‐*γ* treatment (1000 U mL^−1^) for the last 24 h. Cells were then washed twice with PBS, scraped in 0.5 mL of PBS, pelleted and lysed in 50 µL of lysis buffer (50 mm HEPES‐NaOH pH 7.4, 100 mm NaCl, 5 mm EDTA, 1% Triton X‐100 [v/v], 0.5% deoxycholate [w/v], protease inhibitor cocktail, and phosphatase inhibitor) for 1 h at 4 °C. After lysis, nuclei were removed by centrifugation (3000 × *g*, 10 min), and 50% of lysate was analyzed by SDS‐PAGE and western blot analysis using a LI‐COR Odyssey CLx Imaging System.

### Methyl‐*β*‐Cyclodextrin Treatments

The effect of cholesterol depletion from the PM was studied by treatment of HAP1 cells seeded in 35 mm dishes. Treated cells were incubated with 15 mm M*β*CD for 1 h. Rescued cells were treated with 100 µm M*β*CD for 1 h followed by washing twice with PBS an addition of water‐soluble 250 µm chol for 1h. Treated, rescued, and non‐treated (control) cells were then stimulated with IFN‐*γ* for 20 min. Cells were then washed twice with PBS, scraped in 0.5 mL of PBS, pelleted and lysed in 50 µL of lysis buffer (50 mm HEPES‐NaOH pH 7.4, 100 mm NaCl, 5 mm EDTA, 1% Triton X‐100 [v/v], 0.5% deoxycholate [w/v], protease inhibitor cocktail, and phosphatase inhibitor) for 1 h at 4 °C. After lysis, nuclei were removed by centrifugation (3000 × *g*, 10 min), and 50% of lysate was analyzed by SDS‐PAGE and Western blot analysis using a LI‐COR Odyssey CLx Imaging System.

### PD‐L1 Cell Surface Protein Expression

HAP1, HeLa, and MDA‐MB‐231 cells were seeded in coverslips and chol synthesis was inhibited by treatment with 15 µm of Zg for 48 h. In the last 24 h, cells were stimulated with 1000 U mL^−1^ IFN‐*γ*. Cells were then washed twice with PBS and fixed with 4% PFA for 10 min at room temperature. After three washes with PBS, cells were blocked with 3% BSA in PBS for 45 min and immunostained with 1:100 dilution in 1% BSA in PBS of Alexa Fluor 488‐conjugated anti‐PD‐L1 antibody for 1 h at room temperature. Cell PM was stained with WGA‐Alexa Fluor 647, and coverslips mounted in media containing DAPI. PD‐L1 cell surface expression was quantified with FIJI‐ImageJ software by calculating the PD‐L1 PM signal (PM mask was realized with WGA‐Alexa Fluor 647 staining).

### Interaction‐Dependent Probe Incorporation Mediated by Enzymes

pAz ligation to cell‐surface IFN‐*γ*R2 protein was performed using exogenous LplA^W37V^. HEK cells were grown on glass coverslips to 80% of confluency and transiently transfected with 400 ng of HA‐LAP2‐IFN‐*γ*R2 construct for 24 h. Cell surface labeling of the protein of interest was performed by adding 10 µm LpIAW37V, 200 µm pAz, 1 mm ATP, and 5 mm magnesium acetate heptahydrate in growth medium for 30 min at 37 °C. Negative controls were performed by omitting either pAz or the LpIA^W37V^ ligase in the reaction mixture during cell surface protein labeling. Next, cells were washed three times with PBS and pAz derivatized by copper‐free click chemistry using 5 µm sulfo‐Cy3‐DBCO for 15 min at 37 °C. Finally, cells were fixed with 4% paraformaldehyde at room temperature for 15 min, blocked in blocking buffer, and immunostained using a 1:300 dilution of rabbit anti‐HA antibody followed by incubation with a secondary antibody goat anti‐rabbit Alexa Fluor 488 conjugate. Three rinses with PBS with 0.1% Tween‐20 were applied between each antibody incubation step. Nuclei were counterstained with Hoechst and imaged by fluorescence microscopy using a Zeiss Apotome.2 microscope.

### ID‐PRIME to Detect the Interaction of IFN‐*γ*R1 and IFN‐*γ*R2

HEK cells were grown to 80% confluency on glass coverslips, then transfected with 400 ng of Flag‐LpIA‐IFN‐*γ*R1 and 400 ng of HA‐LAP2‐IFN‐*γ*R2 (wild type or mutant) constructs per dish using FuGENE (Promega) according to the manufacturer's instructions. 24 h after transfection, cells were left untreated or stimulated with IFN‐*γ* (1000 U mL^−1^ for 5 min) and pAz ligation onto LAP2‐IFN‐*γ*R2 performed by applying 200 µm pAz, 1 mm ATP and, 5 mm magnesium acetate heptahydrate in serum‐free DMEM at 37 °C for 60 min. Excess pAz was washed out with three changes of fresh DMEM over 10 min at 37 °C. Next, pAz‐tagged proteins were derivatized using copper‐free click chemistry using 5 µm sulfo‐Cy3‐DBCO for 30 min at 37 °C. Cells were then washed to remove unreacted dye and fixed with 4% paraformaldehyde at room temperature for 15 min. Finally, cells were blocked in blocking buffer for 30 min, and proceed for immunofluorescence labeling using a 1:300 dilution in blocking buffer of rabbit anti‐HA antibodies followed by incubation with a secondary antibody goat anti‐rabbit Alexa Fluor 488 conjugate. Three rinses with PBS with 0.1% Tween‐20 was applied between each antibody incubation step. Confocal images were acquired using a Zeiss Apotome.2. To quantify cell surface interaction‐dependent pAz labeling, ROIs were manually drawn on transfected cells by visually inspecting the anti‐HA immunofluorescence images. Intensities of cell surface pAZ were computed and normalized on HA intensity. Background fluorescence was measured by drawing a ROI on an untransfected cell.

### Statistical Analysis

OriginPro was used for plotting data and statistical analyses. No data were considered outliers. One‐way ANOVA with Bonferroni´s multiple comparison test was used to determine statistical significance. ***p < 0.001; **p < 0.01; **p < 0.05, ns: not significant.

## Conflict of Interest

The authors declare no conflict of interest.

## Authors Contributions

O.M. and J.A.N.‐G. contributed equally to the work. O.M., J.A.N.‐G., A.A., D.C., O.T., and I.R.‐B. designed the study and performed experiments. J.B.d.l.S. performed RICS experiments and analyses, P.B. performed MOPRO analyzes, M.L. performed signal transduction experiments. C.M.B. and C.L. assisted with the realization and interpretation of the experiments, F.‐X.C. supervised, directed the research, and wrote the manuscript. All authors contributed to the writing and revision of the manuscript.

## Supporting information

Supporting Information

## Data Availability

The data that support the findings of this study are available from the corresponding author upon reasonable request.

## References

[1] V. Corradi , B. I. Sejdiu , H. Mesa‐Galloso , H. Abdizadeh , S. Y. Noskov , S. J. Marrink , D. P. Tieleman , Chem. Rev. 2019, 119, 5775. 30758191 10.1021/acs.chemrev.8b00451 PMC6509647

[2] a) J. B. de la Serna , G. J. Schutz , C. Eggeling , M. Cebecauer , Front. Cell Dev. Biol. 2016, 4, 106; 27747212 10.3389/fcell.2016.00106 PMC5040727

[3] K. Simons , J. L. Sampaio , Cold Spring Harbor Perspect. Biol. 2011, 3, a004697. 10.1101/cshperspect.a004697 PMC3179338 21628426

[4] G. van Meer , D. R. Voelker , G. W. Feigenson , Nat. Rev. Mol. Cell Biol. 2008, 9, 112. 18216768 10.1038/nrm2330 PMC2642958

[5] E. Sezgin , I. Levental , S. Mayor , C. Eggeling , Nat. Rev. Mol. Cell Biol. 2017, 18, 361. 28356571 10.1038/nrm.2017.16 PMC5500228

[6] J. A. Nieto‐Garai , M. Lorizate , F. X. Contreras , Biochim. Biophys. Acta, Biomembr. 2021, 1864, 183813. 34748743 10.1016/j.bbamem.2021.183813

[7] M. Lorizate , O. Terrones , J. A. Nieto‐Garai , I. Rojo‐Bartolomé , D. Ciceri , O. Morana , J. Olazar‐Intxausti , A. Arboleya , A. Martin , M. Szynkiewicz , M. Calleja‐Felipe , J. B. de la Serna , F.‐X. Contreras , Small Methods 2021, 5, 2100430. 10.1002/smtd.202100430 34928061

[8] T. Harayama , H. Riezman , Nat. Rev. Mol. Cell Biol. 2018, 19, 281. 29410529 10.1038/nrm.2017.138

[9] J. H. Lorent , B. Diaz‐Rohrer , X. Lin , K. Spring , A. A. Gorfe , K. R. Levental , I. Levental , Nat. Commun. 2017, 8, 1219. 29089556 10.1038/s41467-017-01328-3 PMC5663905

[10] M. Hundt , Y. Harada , L. De Giorgio , N. Tanimura , W. Zhang , A. Altman , J. Immunol. 2009, 183, 1685. 19592663 10.4049/jimmunol.0803921 PMC2782658

[11] a) I. Levitan , D. K. Singh , A. Rosenhouse‐Dantsker , Front. Physiol. 2014, 5, 65; 24616704 10.3389/fphys.2014.00065 PMC3935357

[12] F. X. Contreras , A. M. Ernst , P. Haberkant , P. Bjorkholm , E. Lindahl , B. Gonen , C. Tischer , A. Elofsson , G. von Heijne , C. Thiele , R. Pepperkok , F. Wieland , B. Brugger , Nature 2012, 481, 525. 22230960 10.1038/nature10742

[13] a) F. Mollinedo , C. Gajate , Adv. Biol. Regul. 2015, 57, 130; 25465296 10.1016/j.jbior.2014.10.003

[14] C. M. Blouin , Y. Hamon , P. Gonnord , C. Boularan , J. Kagan , C. V. de Lesegno , R. Ruez , S. Mailfert , N. Bertaux , D. Loew , C. Wunder , L. Johannes , G. Vogt , F. X. Contreras , D. Marguet , J. L. Casanova , C. Gales , H. T. He , C. Lamaze , Cell 2016, 166, 920. 27499022 10.1016/j.cell.2016.07.003

[15] C. M. Blouin , C. Lamaze , Front. Immunol. 2013, 4, 267. 24027571 10.3389/fimmu.2013.00267 PMC3760442

[16] J. L. Mendoza , N. K. Escalante , K. M. Jude , J. S. Bellon , L. Su , T. M. Horton , N. Tsutsumi , S. J. Berardinelli , R. S. Haltiwanger , J. Piehler , E. G. Engleman , K. C. Garcia , Nature 2019, 567, 56. 30814731 10.1038/s41586-019-0988-7 PMC6561087

[17] A. Garcia‐Diaz , D. S. Shin , B. H. Moreno , J. Saco , H. Escuin‐Ordinas , G. A. Rodriguez , J. M. Zaretsky , L. Sun , W. Hugo , X. Wang , G. Parisi , C. P. Saus , D. Y. Torrejon , T. G. Graeber , B. Comin‐Anduix , S. Hu‐Lieskovan , R. Damoiseaux , R. S. Lo , A. Ribas , Cell Rep. 2017, 19, 1189. 28494868 10.1016/j.celrep.2017.04.031 PMC6420824

[18] A. Kalbasi , A. Ribas , Nat. Rev. Immunol. 2020, 20, 25. 31570880 10.1038/s41577-019-0218-4 PMC8499690

[19] a) L. Ni , J. Lu , Cancer Med. 2018, 7, 4509; 30039553 10.1002/cam4.1700 PMC6143921

[20] J. W. Moss , D. P. Ramji , World J. Exp. Med. 2015, 5, 154. 26309816 10.5493/wjem.v5.i3.154 PMC4543809

[21] K. M. Pollard , D. M. Cauvi , C. B. Toomey , K. V. Morris , D. H. Kono , Discovery Med. 2013, 16, 123. PMC3934799 23998448

[22] C. Thiele , M. J. Hannah , F. Fahrenholz , W. B. Huttner , Nat. Cell Biol. 2000, 2, 42. 10620806 10.1038/71366

[23] P. Haberkant , O. Schmitt , F. X. Contreras , C. Thiele , K. Hanada , H. Sprong , C. Reinhard , F. T. Wieland , B. Brugger , J. Lipid Res. 2008, 49, 251. 17906222 10.1194/jlr.D700023-JLR200

[24] J. A. Nieto‐Garai , A. Arboleya , S. Otaegi , J. Chojnacki , J. Casas , G. Fabrias , F. X. Contreras , H. G. Krausslich , M. Lorizate , Adv. Sci. 2021, 8, 2003468. 10.1002/advs.202003468 PMC7856888 33552873

[25] J. G. Cordero , M. L. Juarez , Y. M. J. A. Gonzalez , L. C. Barron , B. G. Castaneda , PLoS One 2014, 9, e90704.24643062

[26] M. Garcia‐Marcos , S. Pochet , S. Tandel , U. Fontanils , E. Astigarraga , J. A. Fernandez‐Gonzalez , A. Kumps , A. Marino , J. P. Dehaye , Biochim. Biophys. Acta 2006, 1758, 796. 16842738 10.1016/j.bbamem.2006.05.008

[27] J. Fantini , F. J. Barrantes , Front. Physiol. 2013, 4, 31. 23450735 10.3389/fphys.2013.00031 PMC3584320

[28] A. N. Bukiya , A. M. Dopico , J. Lipid Res. 2017, 58, 1044. 28420706 10.1194/jlr.R073452 PMC5454519

[29] D. T. Jones , W. R. Taylor , J. M. Thornton , FEBS Lett. 1994, 339, 269. 8112466 10.1016/0014-5793(94)80429-x

[30] J. D. Bergstrom , M. M. Kurtz , D. J. Rew , A. M. Amend , J. D. Karkas , R. G. Bostedor , V. S. Bansal , C. Dufresne , F. L. VanMiddlesworth , O. D. Hensens , Proc. Natl. Acad. Sci. U. S. A. 1993, 90, 80. 8419946 10.1073/pnas.90.1.80 PMC45603

[31] Y. Miyake , Y. Kozutsumi , S. Nakamura , T. Fujita , T. Kawasaki , Biochem. Biophys. Res. Commun. 1995, 211, 396. 7794249 10.1006/bbrc.1995.1827

[32] E. Garcia , J. B. de la Serna , Methods 2018, 85, 140. 10.1016/j.ymeth.2018.03.008 29605734

[33] M. Compte , S. L. Harwood , I. G. Munoz , R. Navarro , M. Zonca , G. Perez‐Chacon , A. Erce‐Llamazares , N. Merino , A. Tapia‐Galisteo , A. M. Cuesta , K. Mikkelsen , E. Caleiras , N. Nunez‐Prado , M. A. Aznar , S. Lykkemark , J. Martinez‐Torrecuadrada , I. Melero , F. J. Blanco , J. B. de la Serna , J. M. Zapata , L. Sanz , L. Alvarez‐Vallina , Nat. Commun. 2018, 9, 4809. 30442944 10.1038/s41467-018-07195-w PMC6237851

[34] a) L. Scipioni , M. Di Bona , G. Vicidomini , A. Diaspro , L. Lanzano , Commun. Biol. 2018, 1, 10; 30271897 10.1038/s42003-017-0010-6 PMC6053083

[35] a) S. A. Slavoff , D. S. Liu , J. D. Cohen , A. Y. Ting , J. Am. Chem. Soc. 2011, 133, 19769; 22098454 10.1021/ja206435e PMC3547671

[36] C. Uttamapinant , M. I. Sanchez , D. S. Liu , J. Z. Yao , A. Y. Ting , Nat. Protoc. 2013, 8, 1620. 23887180 10.1038/nprot.2013.096 PMC4892701

[37] a) E. Alspach , D. M. Lussier , R. D. Schreiber , Cold Spring Harbor Perspect. Biol. 2019, 11; 10.1101/cshperspect.a028480 PMC6396335 29661791

[38] K. Mimura , J. L. Teh , H. Okayama , K. Shiraishi , L. F. Kua , V. Koh , D. T. Smoot , H. Ashktorab , T. Oike , Y. Suzuki , Z. Fazreen , B. R. Asuncion , A. Shabbir , W. P. Yong , J. So , R. Soong , K. Kono , Cancer Sci. 2018, 109, 43. 29034543 10.1111/cas.13424 PMC5765310

[39] C. Sun , R. Mezzadra , T. N. Schumacher , Immunity 2018, 48, 434. 29562194 10.1016/j.immuni.2018.03.014 PMC7116507

[40] P. Bjorkholm , A. M. Ernst , M. Hacke , F. Wieland , B. Brugger , G. von Heijne , Biochim. Biophys. Acta 2014, 1838, 2066. 24796501 10.1016/j.bbamem.2014.04.026

[41] J. Kyte , R. F. Doolittle , J. Mol. Biol. 1982, 157, 105. 7108955 10.1016/0022-2836(82)90515-0

[42] J. H. Lorent , I. Levental , Chem. Phys. Lipids 2015, 192, 23. 26241883 10.1016/j.chemphyslip.2015.07.022

[43] a) S. Wilmes , M. Hafer , J. Vuorio , J. A. Tucker , H. Winkelmann , S. Lochte , T. A. Stanly , K. D. P. Prieto , C. Poojari , V. Sharma , C. P. Richter , R. Kurre , S. R. Hubbard , K. C. Garcia , I. Moraga , I. Vattulainen , I. S. Hitchcock , J. Piehler , Science 2020, 367, 643; 32029621 10.1126/science.aaw3242 PMC8117407

[44] a) J. M. Mackenzie , A. A. Khromykh , R. G. Parton , Cell Host Microbe 2007, 2, 229; 18005741 10.1016/j.chom.2007.09.003

[45] I. Okoye , A. Namdar , L. Xu , N. Crux , S. Elahi , Oncotarget 2017, 8, 98215. 29228684 10.18632/oncotarget.21003 PMC5716724

[46] J. K. Liao , U. Laufs , Annu. Rev. Pharmacol. Toxicol. 2005, 45, 89. 15822172 10.1146/annurev.pharmtox.45.120403.095748 PMC2694580

[47] D. J. Allsup , A. S. Kamiguti , K. Lin , P. D. Sherrington , Z. Matrai , J. R. Slupsky , J. C. Cawley , M. Zuzel , Cancer Res. 2005, 65, 7328. 16103084 10.1158/0008-5472.CAN-03-1563

[48] I. Domazet , B. J. Holleran , S. S. Martin , P. Lavigne , R. Leduc , E. Escher , G. Guillemette , J. Biol. Chem. 2009, 284, 11922. 19276075 10.1074/jbc.M808113200 PMC2673261

[49] A. Moll , A. Hildebrandt , H. P. Lenhof , O. Kohlbacher , J. Comput.‐Aided Mol. Des. 2005, 19, 791. 16470421 10.1007/s10822-005-9027-x

[50] T. Gaillard , J. Chem. Inf. Model. 2018, 58, 1697. 29989806 10.1021/acs.jcim.8b00312

[51] I. H. Moal , R. A. G. Chaleil , P. A. Bates , Methods Mol. Biol. 2018, 1764, 413. 29605931 10.1007/978-1-4939-7759-8_27

[52] a) C. UniProt , Nucleic Acids Res. 2019, 47, D506; 30395287 10.1093/nar/gky1049 PMC6323992

[53] a) B. N. L. Fu , Z. Zhu , S. Wu , W. Li , Bioinformatics 2012, 28, 3150; 23060610 10.1093/bioinformatics/bts565 PMC3516142

