# Supplementary material for: Identification of a New Cholesterol‐Binding Site within the IFN‐γ Receptor that is Required for Signal Transduction
Source: Adv Sci (Weinh). 2022 Feb 15;9(11):2105170. doi: 10.1002/advs.202105170 (PMC9008429; doi:10.1002/advs.202105170)
Supplement: Supplementary file 1 — Supporting Information [file ADVS-9-2105170-s001.pdf]

ADVANCED SCIENCE
Open Access

Supporting Information

for *Adv. Sci.*, DOI 10.1002/advs.202105170

Identification of a New Cholesterol-Binding Site within the IFN-γ Receptor that is Required for Signal Transduction

*Ornella Morana, Jon Ander Nieto-Garai, Patrik Björkholm, Jorge Bernardino de la Serna, Oihana Terrones, Aroa Arboleya, Dalila Ciceri, Iratxe Rojo-Bartolomé, Cédric M. Blouin, Christophe Lamaze, Maier Lorizate and Francesc-Xabier Contreras**

ADVANCED SCIENCE

Open Access

Supporting Information

for *Adv. Sci.,* DOI: 10.1002/advs.202105170

Identification of a New Cholesterol-Binding Site within the IFN-γ Receptor that is Required for Signal Transduction

*Ornella Morana, Jon Ander Nieto-Garai, Patrik Björkholm, Jorge Bernardino de la Serna, Oihana Terrones, Aroa Arboleya, Dalila Ciceri, Iratxe Rojo-Bartolomé, Cédric M. Blouin, Christophe Lamaze, Maier Lorizate, and Francesc-Xabier Contreras**

SUPPLEMENTAL INFORMATION

Supplemental Figures

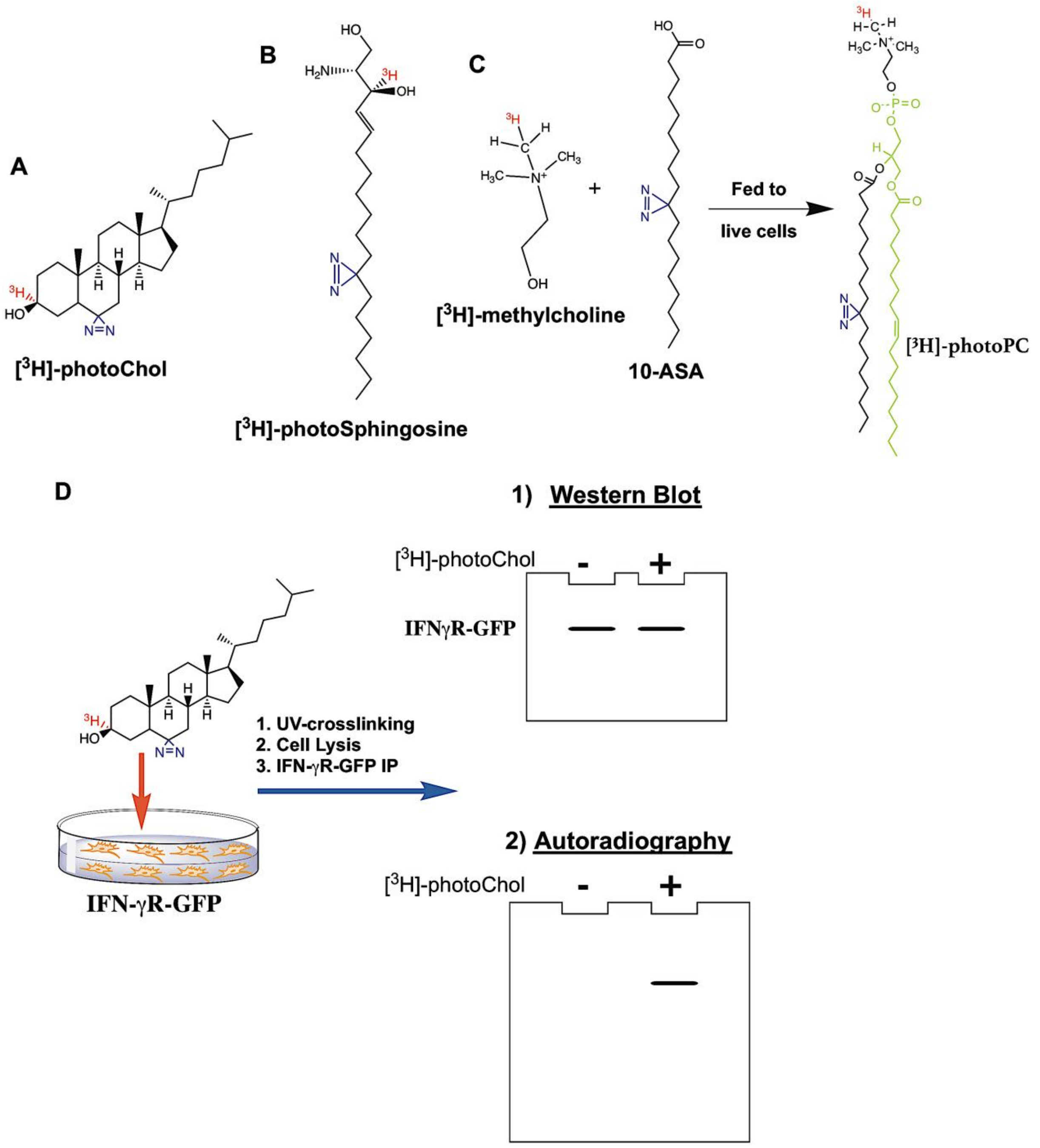

Figure S1. Bifunctional lipid probes and workflow used for *in vivo* photoaffinity binding studies. (A-B) Lipid nanodomains markers. Tritiated and photoactivatable cholesterol and Sph analogues. The photoactivatable diazirine group is highlighted in blue, whereas the ^{3}H is depicted in red. **(C)** Molecular structure of tritiated methyl-choline and photoactivatable 10-ASA probe. The photolabile diazirine group and the tritiated hydrogen are highlighted in blue and red, respectively. In green, molecules provided by the endogenous PC biosynthesis pathway.

D) Schematic representation of the *in vivo* photoactivation cholesterol-binding assay. Cells expressing the IFN-γR-GFP protein subunit to investigate are treated with the bifunctional cholesterol probe, followed by UV-crosslinking, GFP immunoprecipitation, and interaction detected by Western blot and digital autoradiography.

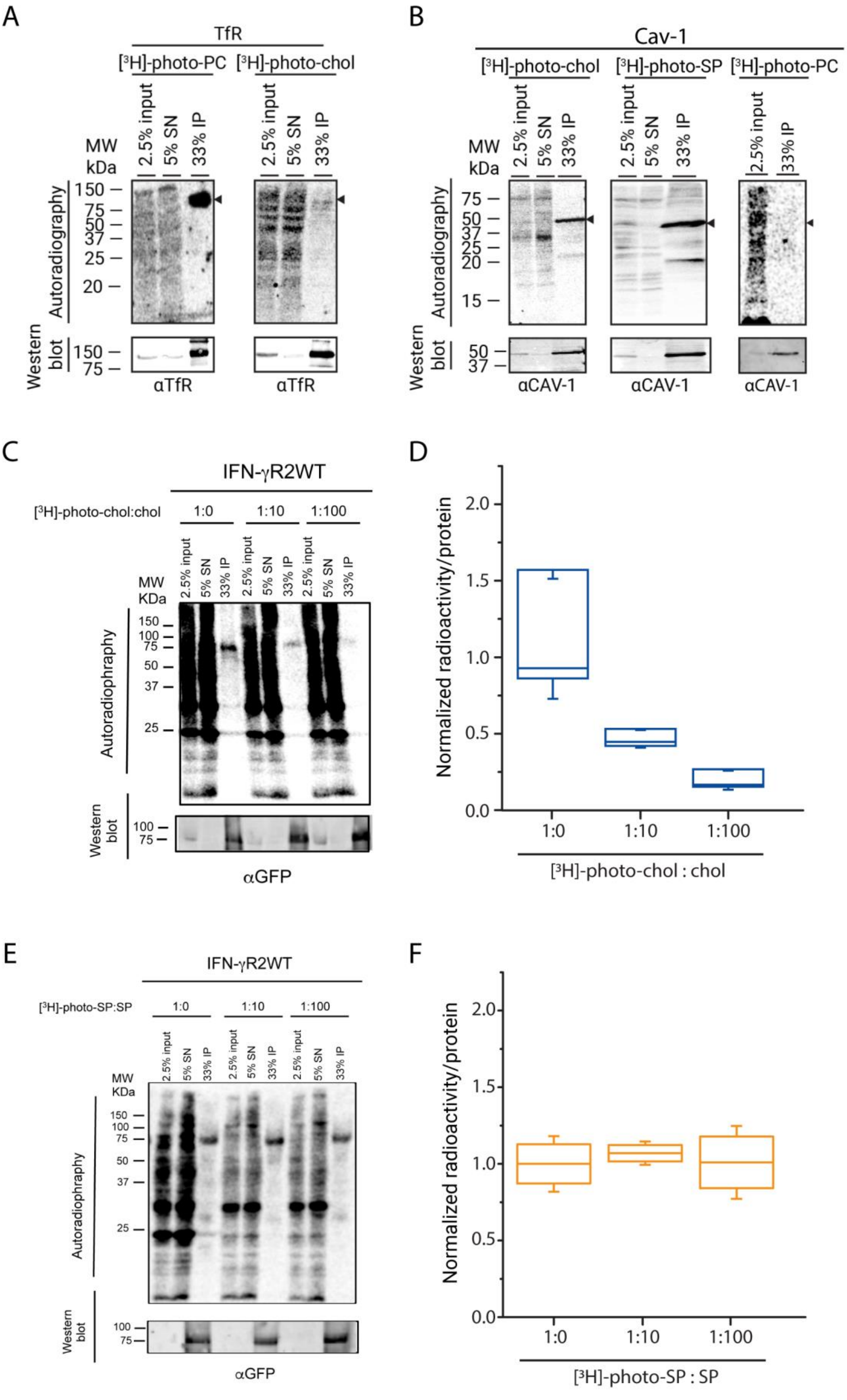
A
TfR
[3H]-photo-PC
[3H]-photo-chol
2.5% input
5% SN
33% IP
MW
kDa
150
75
50
37
25
20
Autoradiography
Western blot
αTfR
B
Cav-1
[3H]-photo-chol
[3H]-photo-SP
[3H]-photo-PC
15
αCAV-1
C
IFN-γR2WT
[3H]-photo-chol:chol
1:0
1:10
1:100
KDa
100
Autoradiophraphy
αGFP
D
Normalized radioactivity/protein
2.0
1.5
1.0
0.5
0.0
[3H]-photo-chol : chol
E
[3H]-photo-SP:SP
F
[3H]-photo-SP : SP

Figure S2. Photolabeling experiments of non-lipid nanodomain marker (transferrin receptor) and lipid nanodomain marker (Cav-1) with bifunctional lipids and competition experiments between bifunctional analogues and cold lipids in living cells. (A,B) *In vivo* photoaffinity labeling of TfR and Cav-1 using tritiated and photolabile lipid probes. (A) Cells treated either with 100 μCi (3 μM) of the bifunctional chol or 50 μCi [^{3}H]-choline combined with 10-ASA (100 μM) for 6 h. Cells were UV-irradiated, lysed, TfR protein subjected to immunoprecipitation and input, supernatant (SN), and immunoprecipitation (IP) analyzed by Western blot and digital autoradiography (n = 3). (B) CHO cells transiently expressing Cav-1-GFP were treated either with 100 μCi (3 μM) or 60 μCi (2 μM) of the bifunctional chol and SP analogues for 6 h, respectively, or with 50 μCi [^{3}H]-choline combined with 10-ASA (100 μM) for 6 h. Cells were UV-irradiated, lysed, Cav-1-GFP protein subjected to immunoprecipitation using GFP antibodies and input, supernatant (SN), and immunoprecipitation (IP) analyzed by Western blot against Cav-1 and digital autoradiography (n = 3). (C) Cross-linking of [^{3}H]-photo-chol (3 μM) to IFN-γR2-GFP in the presence of increasing amounts of competing native chol in the culture media. Cells were UV-irradiated, lysed and handled as described in Figure 1B. (D) Quantification of 3 independent sterol cross-linking experiments as described in (C). Shown are mean + SD. N=3 samples/condition. (F) Cross-linking of [^{3}H]-photo-SP (2 μM) to IFN-γR2 in the presence of increasing amounts of competing native Sph (precursor of SP) in the culture media. Cells were UV-irradiated, lysed, and handled as described in Figure 1B (n = 3). (G) Quantification of 3 independent sphingolipid cross-linking experiments as described in (F). Shown are mean + SD. N=3 samples/condition.

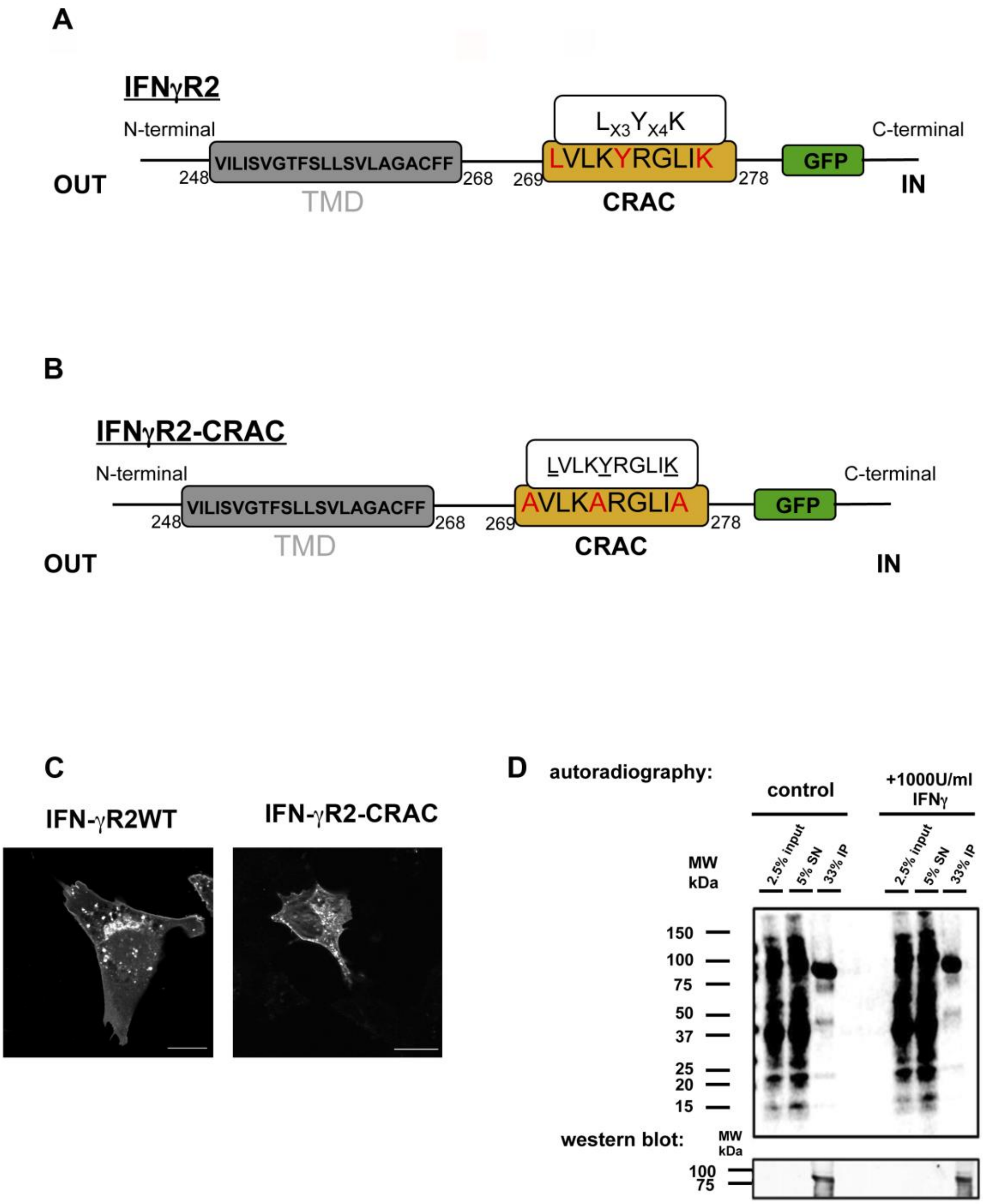

Figure S3. Role of previously described chol-binding motif in IFN-γR2-chol interaction *in vivo*. (A) CRAC domain localization in the C-terminal juxtamembrane region of the IFN-γR2 protein. Amino acids forming the CRAC motif are depicted in red. (B), IFN-γR2-CRAC-GFP construct. Mutated amino acids from the CRAC motif are highlighted in red along with the yellow box. (C) Fluorescence microscopy images of IFN-γR2WT and IFN-γR2-CRAC-GFP tagged constructs distribution in CHO cells (n = 3 independent experiments). Scale bar, 20 μm. (D) *In vivo* photoaffinity experiments of IFN-γR2-CRAC protein with the bifunctional cholesterol probe. CHO cells transiently expressing IFN-γR1WT-RLuc and IFN-γR2-CRAC-GFP proteins were treated with 100 μCi (3 μM) of the bifunctional chol lipid probe for 6 h.

Before ultraviolet irradiation, cells were treated for 5 min with IFN-γ (1000 U/ml) or vehicle. Finally, cells were lysed and handled as described in Figure S2 (n = 3 independents experiments).

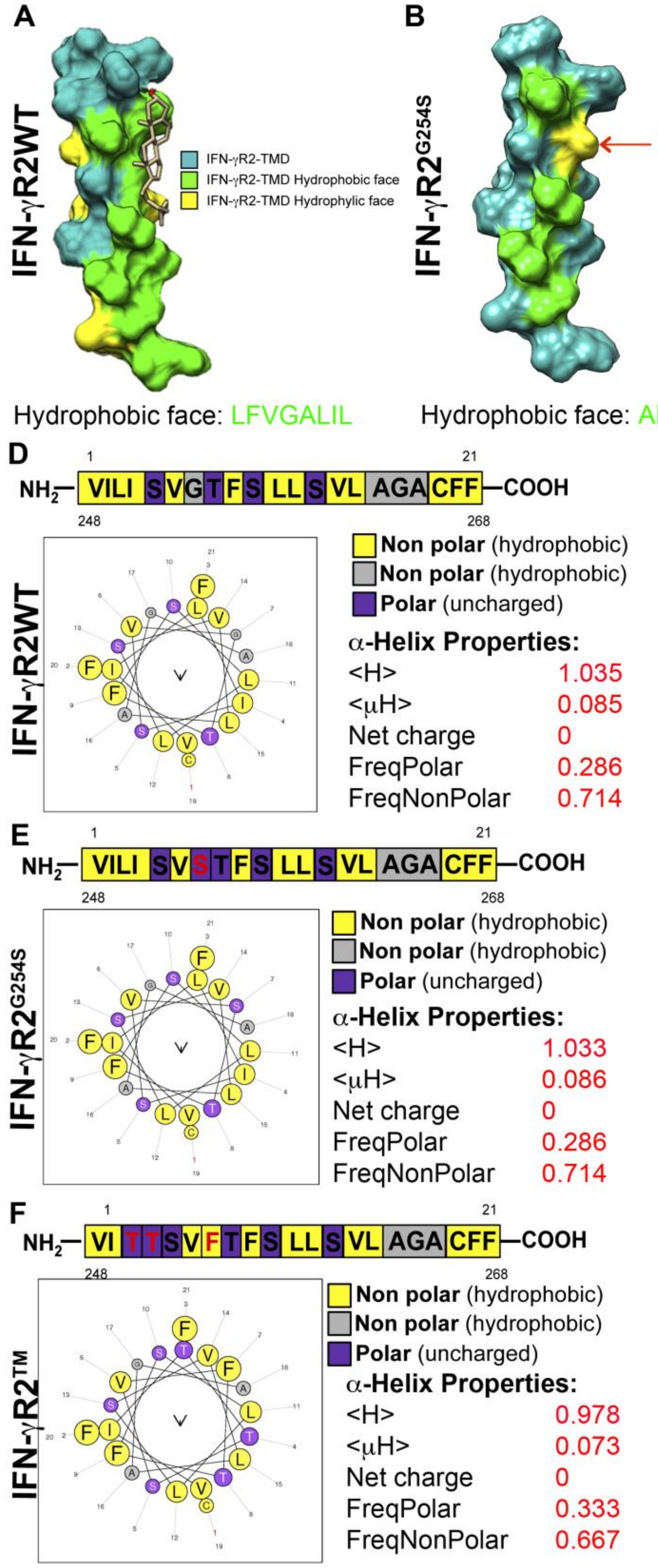

G

	Accessible Surface Area (ASA) A^2
IFN-γR2WT	608.68
IFN-γR2^{G254S}	612.57
IFN-γR^{TM}	604.07

Figure S4. Physiochemical analysis of IFN-γR2TMD wild type and mutants. (A-C**)** 3D minimal energy structures of wild-type IFN-γR2TMD with cholesterol bound, IFN-γR2^{G254S} and IFN-γR2^{TM} mutants. In yellow, marked by a red arrow, the mutation sites. Amino acids forming the hydrophobic phase, containing the chol-binding motif, are depicted in green Amino acids forming the hydrophobic face are named. D-F) Amino acid sequence, α-helix properties,

and two-dimensional clockwise helical projection of wild-type IFN-γR2TMD, IFN-γR2^{G254S} and IFN-γR2^{TM} mutants. The one-letter code size is proportional to amino acid volume. Color-coding indicates amino acid characteristics. Yellow, non-polar hydrophobic; grey, alanine or glycine; purple, uncharged polar residues. Mutated residues are depicted in red in the amino acid sequence. The central symbol represents the hydrophobic moment, while side chains protrude from a circle every 100º. (G) Accessible Surface Area (ASA) values for wild-type IFN-γR2TMD, IFN-γR2^{G254S} and IFN-γR2^{TM} mutants.

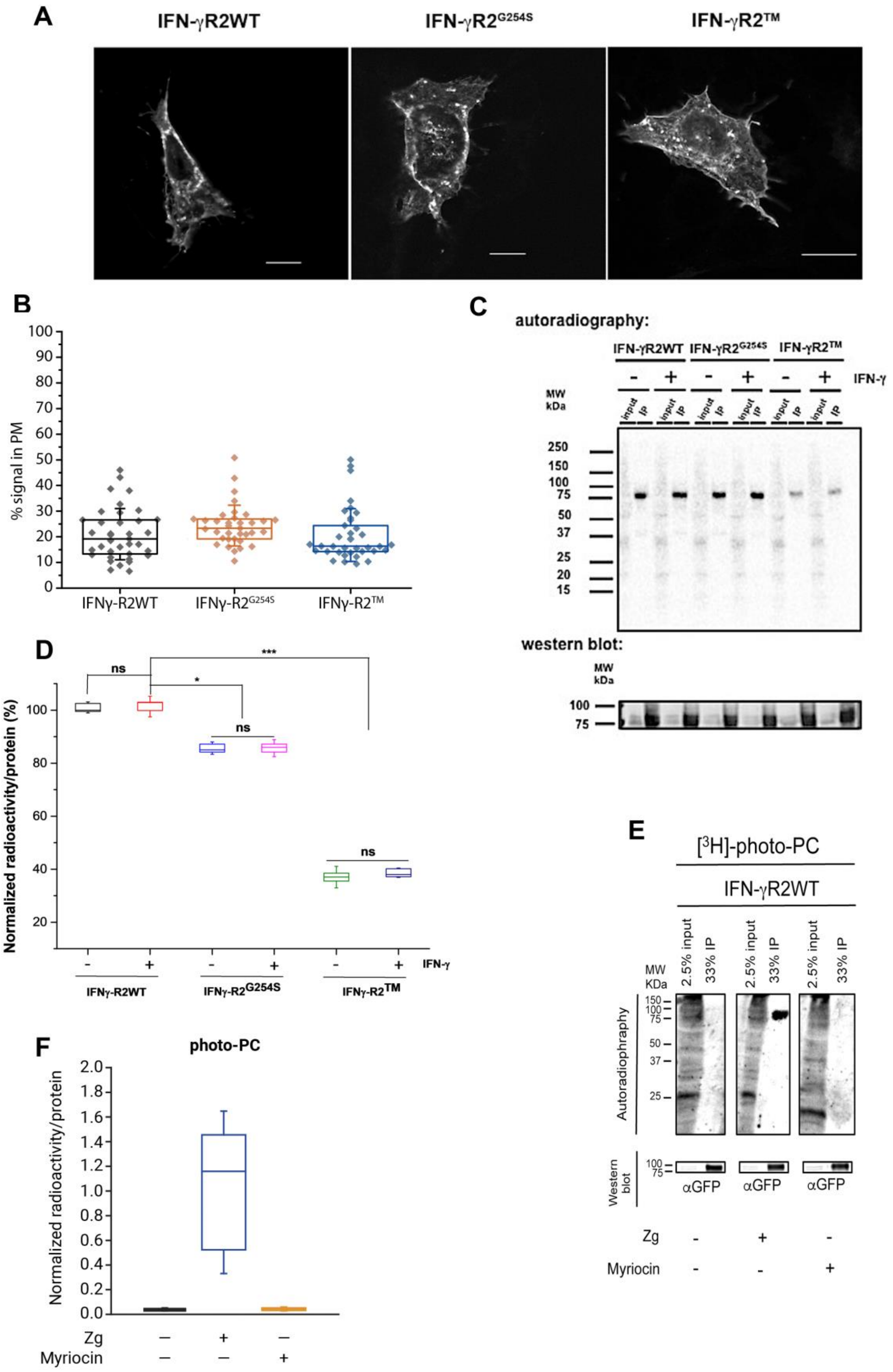

Figure S5. Validation of the novel chol-binding domain localized within the IFN-γR2TMD. (A) Cellular fluorescence localization of transiently express full-length GFP tagged IFN-γR2WT, IFN-γR2^{G254S}, and IFN-γR2^{TM} proteins in CHO cells. Scale bar, 20μm. (B) PM

localization of the wild-type and mutant receptors. (C) *In vivo* photoaffinity labeling of IFN-γR2 wild type and mutants using tritiated and photolabile cholesterol. CHO cells transiently expressing IFN-γR1-RLuc with IFN-γR2WT-GFP or IFN-γR2-GFP mutants were treated with 100 μCi (3 μM) of the bifunctional chol lipid for 6 h. Before ultraviolet irradiation, cells were treated for 5 min with IFN-γ (1000 U/ml) or vehicle. Finally, cells were lysed, subjected to immunoprecipitation against the GFP epitope and input, supernatant (SN), and immunoprecipitation (IP) analyzed by Western blot and digital autoradiography (n = 3 independents experiments). D) Quantification of immunoprecipitated radioactivity for chol binding. Data represent the mean of n = 3 independent experiments ± SD. P-values of one-way ANOVA Bonferroni's multiple comparison test (***p<0.001, *p<0.1, ns = not significant) is given. The line on each of the boxes represents the median for that particular data set. E) *In vivo* photoaffinity binding of PC to IFN-γR2WT in $HAP1^{IFN\text{-}\gamma R2KO}$ cells treated with 15 μM Zg or 25 μM myriocin for 48 h in the presence of 50 μCi (2 μM) [^{3}H]-choline and 100 μM 10-ASA for the last 6 h. F) Quantification of immunoprecipitated radioactivity/protein for PC binding (data are the mean ± SD; n = 3 independent experiments).

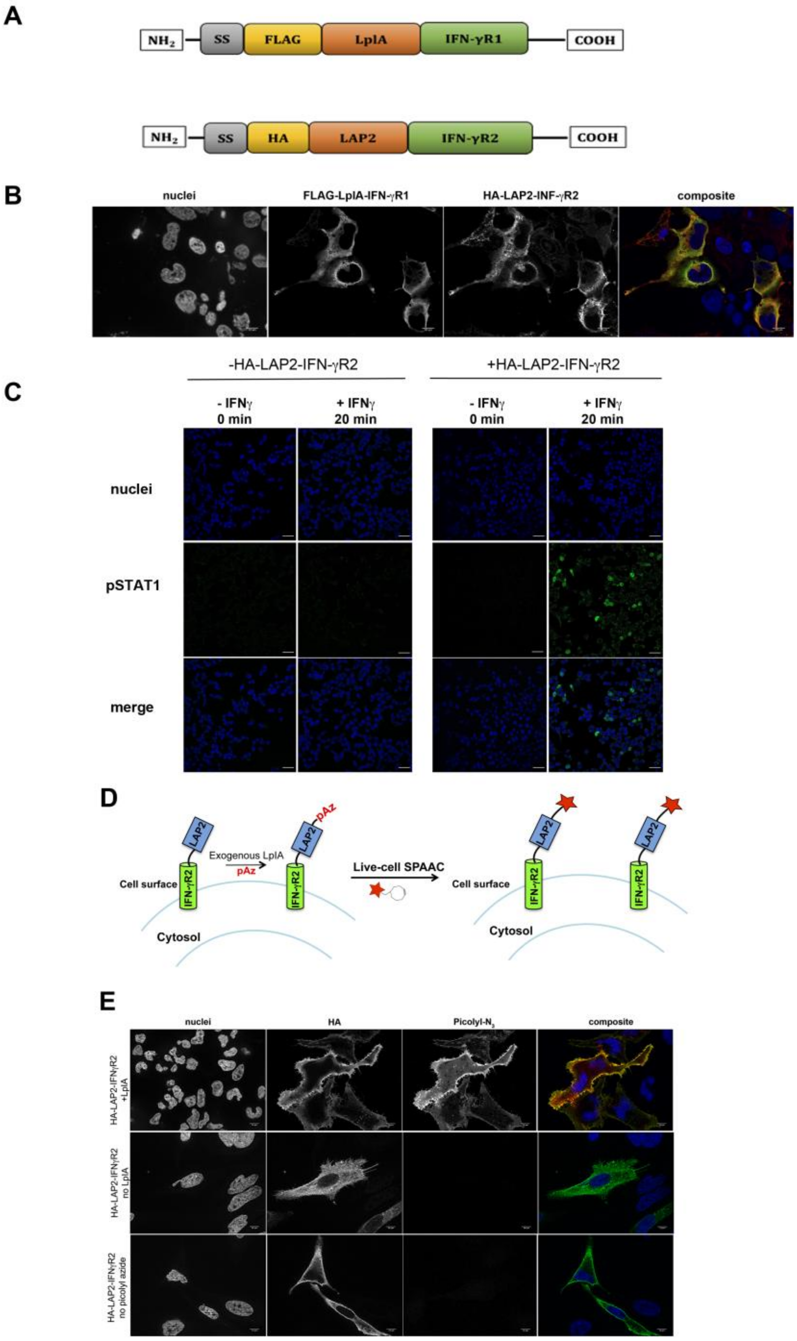

Figure S6. Characterization of IFN-γR1 and IFN-γR2 constructs for ID-PRIME and proof of principle. (A) Flag-LpIA-IFN-γR1 and HA-LAP2-IFN-γR2 constructs. The amino acid sequence of IFN-γR1 was obtained from Uniprot. A Flag tag was inserted between the IFN-γR1 signal sequence (SS) and the LpIA ligase. To not compromise protein domain's biological functions or the formation of secondary structures, a series of four Gly_4Ser repeats flexible linkers were introduced, flanking both sides of the LpIA domain and separating the LpIa protein from the IFN-γR1. A similar strategy was followed to design the HA-LAP2-IFN-γR2 constructs. The amino acid sequence of IFN-γR2 was obtained from Uniprot. An HA tag was introduced

after the IFN-γR2 signal sequence (SS) and the LAP2 peptide sequences. As described in A, a repeat series of Gly_4Ser flexible linkers were introduced, flanking the LAP2 domain and separating the LAP2 sequence from IFN-γR2. (B) Immunofluorescence images of transiently expressed Flag-LpIA-IFN-γR1 and HA-LAP2-IFN-γR2 constructs in HEK293T cells (n = 3 independent experiments). Green, Flag-LpIA-IFN-γR1; red, HA-LAP2-IFN-γR2, blue, nucleus. Scale bar, 10 μm. (C) Immunofluorescence images of pSTAT1 nuclear translocation in $HAP1^{IFN\text{-}\gamma R2ko}$ cells not transfected (left panel) or transiently transfected (right panel) with the HA-LAP2-IFN-γR2 construct prior to and 20 min after IFN-γ stimulation (n = 3 independent experiments). Green, pSTAT1; blue, nuclei. (D) Scheme of *in vivo* HA-LAP2-IFN-γR2 (N-terminal LAP2 facing the extracellular milieu) labeling using a purified recombinant LplA protein ($LpIA^{W37V}$) and pAz. In a second step, pAz was fluorescently labeled via copper-free click chemistry using a fluorescently labeled cyclooctyne. (E) Demonstration of cell surface labeling specificity. HEK 293 cells transiently expressing HA-LAP2-IFN-γR2 construct were labeled live with 10 μM $LpIA^{W37V}$, 200 μM pAz, and 1 mM ATP for 20 min at 37 ºC. After two rounds of washing, cells were labeled *in vivo* with 20 μM DBCO-Cy3 for 30 min. Finally, non-reacted probe was washed out, cells fixed using paraformaldehyde, and HA-LAP2-IFN-γR2 protein detected by immunofluorescence (n = 3 independent experiments). Negative controls are shown with no exogenous addition of the $LpIA^{W37V}$ enzyme or pAz omission during the ID-PRIME step. Green, IFN-γR2; red; pAz; blue, nucleus. Scale bar, 10 μm.

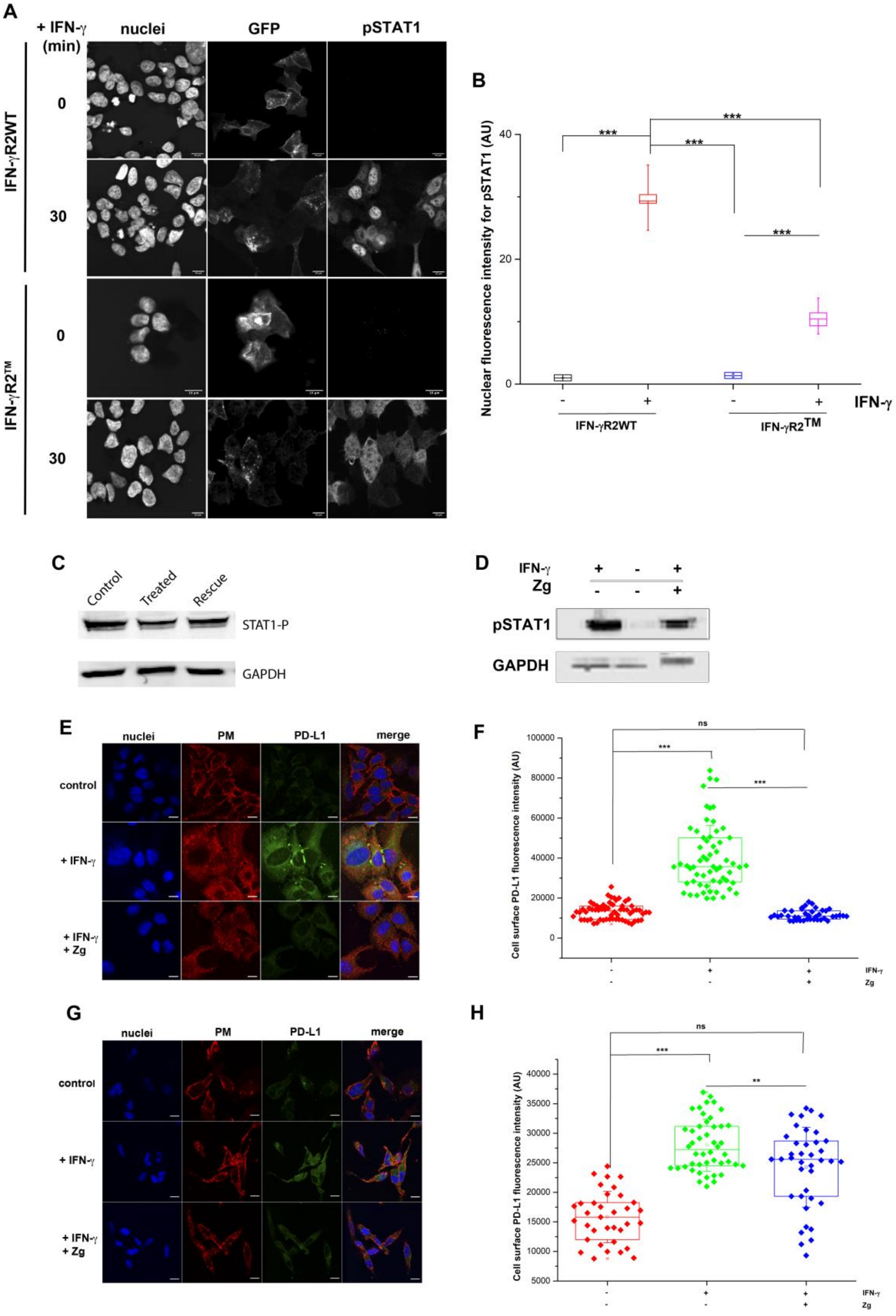

Figure S7. Chol binding is required for IFN-γR transmembrane signal activation and PD-L1 cell surface exposure in response to IFN-γ stimulation. (A) Immunofluorescence images of pSTAT1 nuclear translocation in cells expressing full-length IFN-γR2WT or IFN-γR2^{TM}

proteins before and following 30 min of IFN-γ addition (n = 4 independent experiments). Scale bar, 20 μm. (B) Quantification of pSTAT1 nuclear translocation in $HAP1^{IFN\text{-}\gamma R2KO}$ cells expressing full-length IFN-γR2WT or $IFN\text{-}\gamma R2^{TM}$ proteins before and after 30 min of IFN-γ stimulation. Data represent the mean of n = 3 independent experiments ± SD. n = 50 cells/condition. The line on each of the boxes represents the median for that particular data set. (C,D) Cholesterol depletion or synthesis inhibition downregulates STAT1 phosphorylation in HAP1 cells. (C) Immunoblot of STAT1 phosphorylation in HAP1 cells after 1 h MβCD treatment (15 mM), not treated (control), and water-soluble chol rescue (100 μm, 2 h) followed by IFN-γ addition (n = 3 independent experiments). (D) Immunoblot of pSTAT1 in HAP1 cells after 48 h of Zg (15 μM) treatment followed by IFN-γ stimulation (last 24 h) (n = 3 independent experiments). (E**)** Immunofluorescence images of PD-L1 expression in HeLa cells (cervical cancer) treated with 15 μM of Zg (chol synthesis inhibitor) for 48 h and IFN-γ stimulation for the last 24 h. PD-L1 cell surface expression was labeled with a rabbit anti-PD-L1 antibody labeled with Alexa Fluor 488. Nuclei and PM were counterstained with Hoechst and WGA-Alexa Fluor 647, respectively (representative image of n = 3 independent experiments). (F) Quantification of PD-L1 cell surface protein expression in cells treated and handled as described in E. Data represent the mean of n = 3 independent experiments ± SD. (G,H) Immunofluorescence images and quantification of PD-L1 cell surface protein expression in MDA-MB231 (breast cancer) treated and handled as described in E. P-values of one-way ANOVA Bonferroni's multiple comparison test (***$p<0.001$; **$p<0.0$; ns= not significant) is given.

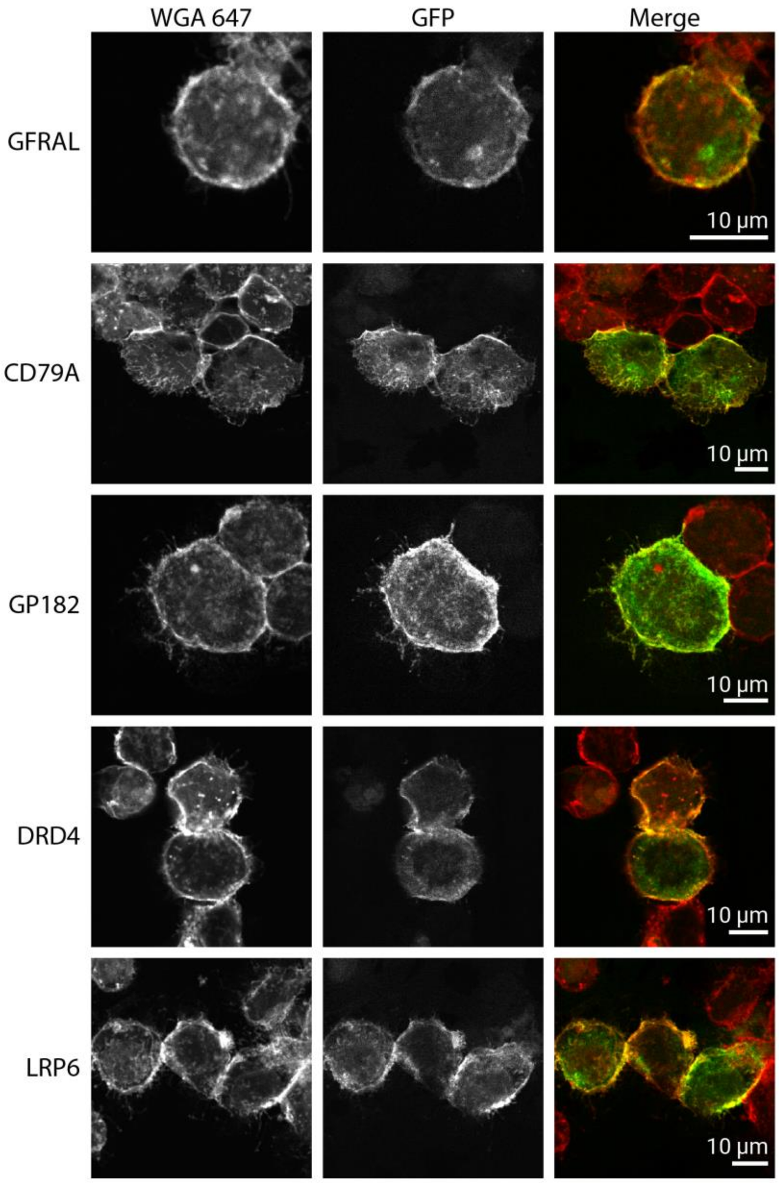

Figure S8. Subcellular localization of candidate chol-binding proteins. HAP1 cells were transiently transfected with GFP-tagged GFRAL, CD79A, GP182, DRD4 and LRP6 proteins. Cells were co-stained with the lectin WGA-AlexaFluor647 a well-validated PM marker (n =3 independent experiments).

Supplementary Table 1. Single-and Multi-span motifs found using MOPRO.

Single-span motif	P-value	Z-value
QXXTIXXAXXXT	0.00	10.08
NXXVIXXAXXXT	0.01	6.97
NXXITXXGXXXL	0.01	6.02
QXXTLXXAXXXI	0.02	5.28
NXXVIXXGXXXL	0.03	4.00
QXXLVXXGXXXI	0.03	3.69
NXXLVXXAXXXI	0.04	3.51
NXXLIXXAXXXV	0.04	3.38
**Multi-span motif**	**P-value**	**Z-value**
NXXIVXXAXXXL	1.00E-04	6.17
QXXILXXAXXXV	6.00E-04	5.05
NXXILXXAXXXL	0.0082	3.48
QXXILXXGXXXV	0.0083	3.72
NXXLLXXGXXXV	0.0096	3.49
NXXILXXGXXXV	0.0146	3.26
NXXLIXXGXXXV	0.0152	3.30
QXXILXXGXXXL	0.0309	2.65
NXXTTXXGXXXV	0.0356	3.65
NXXLLXXGXXXL	0.0499	2.56

Supplementary Table 2. List of chol-binding protein candidates.

50 hits were detected from 8 and 10 signatures for single- and multi-span membrane proteins:

(Q/N)XX(V/I/T/L)XX(G/A)XXX(V/I/T/L)

ID	Name (Single Span Motif)	Motif
Q1LZ86	ABHD6_BOVIN Monoacylglycerol lipase	NXXVIXXGXXXL
Q8R534	ADM1B_MOUSE Disintegrin and metalloproteinase domain-containing protein 1b	NXXLIXXAXXXV
Q07812	BAX_HUMAN Apoptosis regulator	QXXTIXXAXXXT
P0CAN6	CD79A_CANFA B-cell antigen receptor complex-associated protein alpha chain	NXXITXXGXXXL
Q6UXV0	GFRAL_HUMAN GDNF family receptor alpha-like	NXXVIXXAXXXT
P50636	RN19A_MOUSE E3 ubiquitin-protein ligase RNF19A	QXXTLXXAXXXI
Q9QUM4	SLAF1_MOUSE Signaling lymphocytic activation molecule	QXXLVXXGXXXI
Q8IU68	TMC8_HUMAN Transmembrane channel-like protein 8	NXXLVXXAXXXI
Q9H354	YJ001_HUMAN Putative uncharacterized protein PRO1933	NXXLVXXAXXXI
Q96GQ5	CP058_HUMAN UPF0420 protein C16orf58	QXXLVXXAXXXV
Q3TQB2	FXRD1_MOUSE FAD-dependent oxidoreductase domain-containing protein 1	QXXVVXXGXXXL
Q5SZI1	LRAD2_HUMAN Low-density lipoprotein receptor class A domain-containing protein 2	QXXLLXXAXXXT
**ID**	**Name (Multi Span Motif)**	**Motif**
P34969	5HT7R_HUMAN 5-hydroxytryptamine receptor 7	NXXIVXXAXXXL
Q50JE5	ACE_MESAU Angiotensin-converting enzyme	QXXLLXXGXXXL
O95573	ACSL3_HUMAN Long-chain-fatty-acid--CoA ligase 3	NXXLLXXGXXXL
O02666	ADA1D_RABIT Alpha-1D adrenergic receptor	NXXIVXXAXXXL
A2RT91	ANKAR_MOUSE Ankyrin and armadillo repeat-containing protein	QXXILXXAXXXV
Q9NQ90	ANO2_HUMAN Anoctamin-2	QXXIIXXGXXXI
O43861	ATP9B_HUMAN Probable phospholipid-transporting ATPase IIB	QXXILXXGXXXL
Q9WVK0	ATRAP_MOUSE Type-1 angiotensin II receptor-associated protein	NXXILXXGXXXV
Q8CDN1	CC020_MOUSE Uncharacterized protein C3orf20 homolog	NXXLLXXGXXXL
Q599A1	COX1_BALBO Cytochrome c oxidase subunit 1	NXXVTXXAXXXI
Q6NUT3	CS028_HUMAN Uncharacterized MFS-type transporter C19orf28	NXXLLXXGXXXV
Q9NQ40	CT054_HUMAN Uncharacterized protein C20orf54	NXXLLXXGXXXV

A6NN92	CXE1_HUMAN Gap junction epsilon-1 protein	QXXTLXXGXXXI
P21917	DRD4_HUMAN D(4) dopamine receptor	NXXIVXXAXXXL
Q8BLD9	DRD5_MOUSE D(1B) dopamine receptor	NXXIVXXAXXXL
F1N476	ECE2_BOVIN Endothelin-converting enzyme 2	QXXLVXXGXXXL
O75899	GABR2_HUMAN Gamma-aminobutyric acid type B receptor subunit 2	QXXLVXXGXXXL
Q8NFN8	GP156_HUMAN Probable G-protein coupled receptor 156	QXXTIXXGXXXL
O15218	GP182_HUMAN G-protein coupled receptor 182	NXXILXXAXXXL
Q99P91	GPNMB_MOUSE Transmembrane glycoprotein NMB	NXXLIXXGXXXV
A1DWM3	MFSD6_PIG Major facilitator superfamily domain-containing protein 6	NXXTTXXGXXXV
Q9Y5X5	NPFF2_HUMAN Neuropeptide FF receptor 2	NXXILXXAXXXL
P41308	NU4M_DIDMA NADH-ubiquinone oxidoreductase chain 4	NXXILXXGXXXV
P32240	PE2R4_MOUSE Prostaglandin E2 receptor EP4 subtype	QXXILXXAXXXV
Q9UKG4	S13A4_HUMAN Solute carrier family 13 member 4	NXXLLXXGXXXV
Q16348	S15A2_HUMAN Solute carrier family 15 member 2	NXXLLXXGXXXL
A6NIM6	S15AX_HUMAN Peptide/histidine transporter ENSP00000340402	NXXIVXXGXXXI
Q8WMS0	S35A2_CANFA UDP-galactose translocator	QXXILXXAXXXV
A6QL92	S35F5_BOVIN Solute carrier family 35 member F5	NXXLLXXGXXXL
Q5RC98	S38AA_PONAB Putative sodium-coupled neutral amino acid transporter 10	QXXIVXXAXXXV
Q3SYU3	S39A1_BOVIN Zinc transporter ZIP1	QXXILXXGXXXV
Q31125	S39A7_MOUSE Zinc transporter SLC39A7	QXXILXXGXXXL
Q4G0N8	S9A10_HUMAN Sodium/hydrogen exchanger 10	NXXILXXAXXXL
Q8WTV0	SCRB1_HUMAN Scavenger receptor class B member 1	QXXLLXXGXXXL
O77760	SOAT1_CERAE Sterol O-acyltransferase 1	QXXILXXGXXXV
Q86WV6	TM173_HUMAN Transmembrane protein 173	QXXLLXXGXXXL
Q8R115	TMM82_MOUSE Transmembrane protein 82	QXXLVXXGXXXL
O88799	ZAN_MOUSE Zonadhesin	NXXLIXXGXXXV
